# Supplementary material for: A Digitally Enabled, Pharmacist service to detecT medicine harms in residential aged care (nursing home) (ADEPT): protocol for a feasibility study
Source: BMJ Open. 2024 Feb 10;14(2):e080148. doi: 10.1136/bmjopen-2023-080148 (PMC10862280; doi:10.1136/bmjopen-2023-080148)
Supplement: Supplementary data [file bmjopen-2023-080148supp001.pdf]

University of South Australia

Consent Form

This project has been approved by the University of South Australia's Human Research Ethics Committee. If you have any ethical concerns about the project, or questions about your rights as a participant, please contact the Executive Officer of this Committee, Tel: +61 8 8302 6330; Email: humanethics@unisa.edu.au

SECTION 1: CONTACT AND PROJECT DETAILS	
Researcher's Full Name	[Chief Investigator]
Contact Details	[Phone number]
Project Number	ADEPT-2022.1.0 (Ethics ID 205098)
Project Title	A Digitally Enabled, Pharmacist service to detecT medicine harms in residential aged care: the ADEPT project

SECTION 2: PARTICIPANT CERTIFICATION

In signing this form, I confirm that:

- I have read the Participant Information Sheet, and the nature and the purpose of the research project has been explained to me. I understand and agree to take part.
- I understand the nature of my involvement in the project.
- I understand that I may not directly benefit from taking part in the project.
- I understand that I can withdraw from the project at any stage and that this will not affect my status now or in the future.
- I understand that data will be stored in the secure settings in the Quality Use of Medicines and Pharmacy Research Centre, University of South Australia. I understand that all paper and electronic records collected as part of the project will be kept for 7 years after final publication of project results.
- I understand that while information gained during the project may be published, I will not be identified and my personal results will remain confidential, unless required by law.
- I understand that data collected from this project may be used for another purpose by the researcher for which ethics approval will be sought.

Participant's Signature	*Printed Name*	*Date*
Legal guardian's Signature (if signing of behalf of participant)	*Printed Name*	*Date*

SECTION 3: RESEARCHER CERTIFICATION

I have explained the study to the participant and/or legal guardian and consider that he/she understand what is involved.

Researcher Signature	*Printed Name*	*Date*

CRICOS Provider No. 00121B
