# Supplementary material for: A Digitally Enabled, Pharmacist service to detecT medicine harms in residential aged care (nursing home) (ADEPT): protocol for a feasibility study
Source: BMJ Open. 2024 Feb 10;14(2):e080148. doi: 10.1136/bmjopen-2023-080148 (PMC10862280; doi:10.1136/bmjopen-2023-080148)
Supplement: Author's manuscript [file bmjopen-2023-080148.draft_revisions.pdf]

BMJ Open

BMJ Open is committed to open peer review. As part of this commitment we make the peer review history of every article we publish publicly available.

When an article is published we post the peer reviewers' comments and the authors' responses online. We also post the versions of the paper that were used during peer review. These are the versions that the peer review comments apply to.

The versions of the paper that follow are the versions that were submitted during the peer review process. They are not the versions of record or the final published versions. They should not be cited or distributed as the published version of this manuscript.

BMJ Open is an open access journal and the full, final, typeset and author-corrected version of record of the manuscript is available on our site with no access controls, subscription charges or pay-per-view fees (http://bmjopen.bmj.com).

If you have any questions on BMJ Open's open peer review process please email info.bmjopen@bmj.com

BMJ Open

A Digitally Enabled, Pharmacist service to detecT medicine harms in residential aged care (ADEPT): protocol for a feasibility study.

Journal:	*BMJ Open*
Manuscript ID	bmjopen-2023-080148
Article Type:	Protocol
Date Submitted by the Author:	22-Sep-2023
Complete List of Authors:	Boord, Monique; University of South Australia, Quality Use of Medicines and Pharmacy Research Centre<br>Brown, Peter; University of New South Wales, Tyree Foundation Institute of Health Engineering (IHealthE) and Graduate School of Biomedical Engineering<br>Soriano, Julian; Tanunda Lutheran Home Inc; SA Pharmacy<br>Meola, Tahlia; University of South Australia, UniSA Clinical and Health Sciences<br>Dumuid, Dorothea ; University of South Australia, Alliance for Research in Exercise, Nutrition and Activity<br>Milte, Rachel; Flinders University, Rehabilitation, Aged and Extended Care<br>Roughead, Elizabeth; University of South Australia, Quality Use of Medicines and Pharmacy Research Centre, UniSA Clinical and Health Sciences<br>Lovell, Nigel; University of New South Wales, Tyree Foundation Institute of Health Engineering (IHealthE) and Graduate School of Biomedical Engineering<br>Stone, Helen; Pharmaceutical Society of Australia<br>Whitehouse, Joseph; HealthyCare Services<br>Janetzki, Jack; University of South Australia, Quality Use of Medicines and Pharmacy Research Centre; University of South Australia, UniSA Clinical and Health Sciences<br>Gebreyohannes, Eyob Alemayehu; University of South Australia, Quality Use of Medicines and Pharmacy Research Centre, UniSA Clinical and Health Sciences; The University of Western Australia, School of Allied Health<br>Lim, Renly; University of South Australia, Quality Use of Medicines and Pharmacy Research Centre, UniSA Clinical and Health Sciences
Keywords:	Adverse events < THERAPEUTICS, Aged, Health Services for the Aged, GERIATRIC MEDICINE, Health policy < HEALTH SERVICES ADMINISTRATION & MANAGEMENT, Patient-Centered Care

For peer review only - http://bmjopen.bmj.com/site/about/guidelines.xhtml

A Digitally Enabled, Pharmacist service to detecT medicine harms in residential aged care (ADEPT): protocol for a feasibility study.

Authors

Monique S Boord[1], Peter Brown[2], Julian Soriano[3,4], Tahlia Meola[5], Dorothea Dumid[6], Rachel Milte[7], Elizabeth E Roughead[1], Nigel H. Lovell[2], Helen Stone[8], Joseph Whitehouse[9], Jack L Janetzki[1,5], Eyob Alemayehu Gebreyohannes[1,10], Renly Lim[1]

Institutions

[1] Quality Use of Medicines and Pharmacy Research Centre, UniSA Clinical and Health Sciences, University of South Australia, Adelaide, Australia

[2] Tyree Foundation Institute of Health Engineering (IHealthE) and Graduate School of Biomedical Engineering, UNSW, Sydney, Australia

[3] Tanunda Lutheran Home, Tanunda, Australia

[4] SA Pharmacy, Adelaide, Australia

[5] UniSA Clinical and Health Sciences, University of South Australia, Adelaide, Australia

[6] Alliance for Research in Exercise, Nutrition and Activity, UniSA Clinical and Health Sciences, University of South Australia, Adelaide, Australia

For peer review only

[7] Caring Futures Institute, College of Nursing and Health Sciences, Flinders University, Adelaide, Australia

[8] Pharmaceutical Society of Australia, Deakin, Australia

[9] HealthyCare Services, Welland, Australia

[10] School of Allied Health, The University of Western Australia, Perth, Australia

Correspondence to:

Dr Monique Boord

Quality Use of Medicines and Pharmacy Research Centre,

UniSA Clinical and Health Sciences,

University of South Australia, Adelaide, Australia

Monique.boord@unisa.edu.au

For peer review only

Abstract

Introduction

This feasibility study aims to develop and test a new model of practice in Australia: using digital technologies to enable pharmacists to monitor early signs and symptoms of medicine-induced harms in residential aged care.

Methods and analysis

Thirty residents will be recruited from an aged care facility in South Australia. The study will be conducted in two phases. In phase 1, the study team will work with aged care software providers and developers of digital technologies (a wearable activity tracker and a sleep tracking sensor) to gather physical activity and sleep data, as well as medication and clinical data from the electronic medication management system and aged care clinical software. Data will be centralised into a cloud-based monitoring platform (TeleClinical Care). The TeleClinical Care (TCC) will be used to create dashboards that will include longitudinal visualisations of changes in residents' health, function, and medicine use over time. In Phase 2, the on-site pharmacist will use the centralised TCC platform to monitor each resident's medicine, clinical, physical activity and sleep data to identify signs of medicine-induced harms over a 12-week period.

A mixed methods process evaluation applying the RE-AIM evaluation framework will be used to assess the feasibility of the service. Outcome measures include service reach, changes in resident symptom scores (measured using the Edmonton Symptom Assessment System), number of medication adverse events detected, changes in physical activity and

For peer review only

sleep, number of pharmacist interventions provided, cost analysis, and proportion of all pharmacists' recommendations implemented at 4-, 8-, and 12-weeks post-baseline period.

Ethics and dissemination

Ethical approval has been obtained from the University of South Australia's Human Research Ethics Committee (205098). Findings will be disseminated through published manuscripts, conference presentations, and reporting to the study funder.

Trial registration number

ACTRN12623000506695

Strengths and limitations of this study

- This study will be the first to develop and implement a digital platform in residential aged care to enable pharmacists to actively monitor medicine-induced harms.
- This study will use objective assessments, including changes in physical activity and sleep, to identify "mild" medicine harms that are traditionally more difficult to detect.
- Participants are recruited from a single residential aged care facility in South Australia and findings may not generalise across all residents living in aged care.

For peer review only

- A potential limitation is lack of compliance with use and wearing of digital technologies by the residents.

For peer review only

Introduction

Approximately eight percent of adults in Australia aged 65 and older reside in aged-care facilities [1], with up to 63% receiving nine or more regular medications [1]. Medicine-related problems contribute to an estimated 250,000 hospital admissions in Australia every year, with an estimated annual cost of $1.4 billion [2]. Medicine-related harms are of significant concern in residential aged care, with up to 27 adverse medicine events occurring per 100 residents per month [3-5]. In Australia, about one in five persons living in residential aged care experience an adverse medicine event every month; of which 83% were potentially preventable [6]. Despite the significant medicine-related harms, most Australian residential aged care facilities have limited capacity to provide clinical pharmacy services; existing services are limited to government funded residential medication management review and quality use of medicine services [7].

The Australian Government, recognising the significance of medicine-related problems in aged care, committed $350 million to improve medication safety in residential aged care through funding for pharmacists [8]. The government investment presents new opportunities for pharmacists to review residents for signs and symptoms of medicine harms more frequently. The present study builds on our recent randomised controlled trial which investigated the effectiveness of ongoing pharmacist assessments compared with usual care

For peer review only

in reducing medicine induced deterioration, frailty, and adverse events across 39 residential aged care facilities in Australia [9, 10]. Our study showed that one in five residents experienced a preventable adverse event (e.g., falls, fractures) every month due to medication use [9]. The pharmacists identified and resolved medicine-related harms and served as an advocate for residents. Further, significant differences in change in cognition from baseline were observed between intervention and control groups [9]. The service ensured pharmacist engagement with residents, rather than just reviewing medicines, which enabled the advocacy [11].

Frequent monitoring of medicine-induced harms is important because medicines can cause mild harm (e.g., sedation, poor balance) that often remains undetected, but can lead to serious adverse events in older people if left unmanaged. For example, commonly prescribed psychotropic medicines can cause sedation and poor cognition that may lead to injurious falls and fractures [12]. An increase of sedative load from two to four over a 12-month period has been associated with an average increase in daily sedentary behaviour of approximately 24 minutes [13]; an increase of 60 minutes per day of sedentary time is associated with a 19% increase in risk of death [14]. Detecting mild, cumulative effects of medicine-induced harms, including changes in physical activity, is likely to be important, however, this can be difficult and labour intensive to measure and monitor.

For peer review only

Harnessing digital technologies to enable active monitoring of medicine-related harms is one potential strategy to enabling proactive monitoring. For pharmacists to effectively monitor harms, they will not only need to review medicines, but also recognise and monitor early signs of medicine-induced harms such as changes in sleep, activity, or cognition [9, 13]. Digital technologies can measure changes in sleep, activity, or cognition objectively, which may enhance effective communication about medicines-related harm across aged care residents' care teams, including to general practitioners (GPs), nurses and care staff, as well as support the recommendations from pharmacists and their acceptance by the team and residents themselves. The TeleClinical-Care (TCC) platform has been used successfully during the COVID-19 pandemic to safely manage more than 8,000 COVID-positive patients as they self-isolated at home [15] and is being used in trials to manage cardiovascular disease and stroke [16].

The ADEPT project aims to develop and implement a digitally enabled, evidence-based, pharmacist service built upon TCC (TCC-ADEPT) to actively detect medicine harms in residential aged care. Specifically, we will:

1. Determine the number and proportion of eligible residents who were willing to participate in the study;

For peer review only

2. Determine changes in resident outcomes, including changes in symptoms as measured using the Edmonton Symptom Assessment System (ESAS) [17], physical activity and sleep (24-hour movement behaviour), and adverse events including falls (both injurious and non-injurious), fractures, delirium, faecal impaction, hospitalisation or emergency department presentation due to adverse medication events, and medication incidents, pre- and post-intervention;
3. Determine the number and types of pharmacist recommendations that resulted following use of the TCC-ADEPT platform, and the number of pharmacist recommendations that were accepted and implemented by GPs and aged care staff;
4. Explore the acceptability of the interventions by residents and family members, aged care staff and GPs;
5. Assess the costs associated with implementing the TCC-ADEPT platform and potential cost impacts to the health and aged care system in wider-spread roll out of the platform;
6. Explore the residential aged care facility's intention to continue to implement the pharmacist service post study funding and whether the service aligns to the facility's mission or sustainability of business model.

For peer review only

We hypothesise that the new pharmacist service is feasible and acceptable to aged care staff, residents and their GPs, and can enhance detection of early signs and symptoms of medicine harms, triggering review and action when required.

Methods and analysis

Study design

The ADEPT project will be conducted in two phases over a 12-month period. Phase one will include gathering data from digital technologies, medication and health records into the TCC platform. Digital technologies will include an activity tracker [18] and a sleep sensor [19]. A pre-post12-week implementation study will constitute phase two of the project. The on-site pharmacist will monitor all participants using the centralised TCC-ADEPT platform, using data to inform resident reviews and to initiate conversations with residents, GPs and aged care staff on medicine, health and quality of life changes for residents.

Patient and public involvement

Aged care residents, representatives and health professionals were directly involved in the development or design of this study. Findings from interviews with residents, carers

For peer review only

and aged care staff from the ReMInDAR trial were used to inform the development and design of this study [20].

Cohort selection

Residents will be considered eligible to participate if they are a permanent resident living in the designated residential aged care facility. Exclusion criteria include receiving palliative or respite care.

Screening and recruitment

Residents will be recruited from an aged care facility in South Australia. Promotional recruitment material (flyers) will be distributed onsite in common areas of the facility one month prior to study commencement. Participant information sheets will be provided to residents deemed potentially eligible by facility staff, and will be given to all residents willing to participate, informing them of the study details and pharmacist service.

Study staff will perform initial screening of residents' care record to identify potentially eligible residents. Screening logs will be kept monitoring residents' recruitment and to enable a description of the study population from which eligible residents have been enrolled. If the resident fulfills inclusion criteria based on the initial screening process, the on-site

For peer review only

pharmacist will invite the resident to participate in the study and written informed consent will be obtained from the resident or their authorised representative.

Baseline data collection

Once written consent is obtained, study staff will collect baseline data from the Resident Care Assessment Record held electronically in Leecare [21] and Medi-Map [22] software, which contains demographic details, comorbidities, medicine use, falls history, and care provided within the aged care facility. Study staff will administer the Edmonton Symptom Assessment Scale (ESAS) [17] and the Montreal Cognitive Assessment (MoCA) [23] at baseline. An observational logbook or diary will be kept to record information not collected in the existing data fields. Table 1 indicates data collected at each data collection time points.

For peer review only

Table 1. Data collection schedule

Variable	Description	Data source	Data collection time points			
			Baseline	4-weeks	8-weeks	12-weeks
**Demographic**	Age, gender	Leecare	✓			
**Medical conditions**	Diagnosis of medical condition	Leecare	✓	✓	✓	✓
**Medications***	Name, strength, dose, frequency of medicine administration	Medi-Map	✓	✓	✓	✓
**Nutritional status**	Swallowing status	Leecare	✓			✓
**Functional ability**	Mobilisation status	Leecare	✓			✓
**Cognitive function**	Montreal Cognitive Assessment	Leecare	✓			✓
**Falls history**	Number of falls and related health outcomes	Leecare	✓			✓
**Vital signs**	Height, weight, blood pressure (systolic and diastolic), respirations, pulse, BGL, SpO2	Leecare	✓	✓	✓	✓
**Pain**	PainChek® score severity across 6 domains	Leecare	✓	✓	✓	✓
**Residential Medication Management Review (RMMR)**	Dates of RMMR provision will be recorded	On-site aged care pharmacist	✓	✓	✓	✓
**Physical activity**	Time spent in light, moderate, vigorous-intensity activity	Fibion SENS device	✓	✓	✓	✓
**Sleep**	Movements during sleep, durations of light, deep and REM sleep	Fibion Emfit device	✓	✓	✓	✓
**Edmonton Symptom Assessment Scale (ESAS)**	Severity across 10 symptoms	Participant or third-party informant review	✓	✓	✓	✓
**Adverse events**	E.g., falls, fracture, delirium, faecal impaction, incontinence	Leecare, Pharmacists,	✓	✓	✓	✓

For peer review only

		aged care staff, residents				
**Incidents**	Medication incidents will be recorded including date/time and severity	Leecare, Pharmacists, aged care staff, residents	✓	✓	✓	✓
**Ambulance call-outs**	Date and reasons will be recorded	Leecare, Pharmacists, aged care staff, residents	✓	✓	✓	✓
**Hospital visits**	Date and reason of emergency department visits or hospital admission will be recorded	Leecare, Pharmacists, aged care staff, residents	✓	✓	✓	✓
**All-cause mortality**	Any deaths in the 12-week study period will be recorded	Aged care staff		✓	✓	✓

Note* data will be collected and exported daily into the TCC-ADEPT platform over the 12-week study period. BGL= Blood glucose level; SpO2= oxygen saturation; REM= rapid eye movement.

Measures

Edmonton Symptom Assessment Scale (ESAS)

The ESAS is a 10-item numeric rating scale assessing self-reported pain, fatigue, nausea, depression, anxiety, drowsiness, shortness of breath, appetite, wellbeing, and sleep [17]. Participants or their carer will rate the severity of each symptom on a scale of 0-10 by circling a number on each scale which best describes their symptoms over the past 24 hours [17]. Higher scores indicate higher symptom burden.

For peer review only

197 Montreal Cognitive Assessment (MoCA)

198 The MoCA is a 1 page, 30-point screening instrument that assesses cognitive domains of memory, language, executive functions, visuospatial skills, calculation, abstraction, attention, concentration, and orientation [23]. A cut off score of 26 has been shown to differentiate mild cognitive impairment or dementia from normal cognition [23]. When scoring, a one-point adjustment is given to those with 12 or less years of formal education [23].

204 Fibion SENS

205 The activity tracker is the Fibion SENS device (SENS Motion®, Copenhagen, Denmark) [18] which is a waterproof 3D triaxial accelerometer (4.7 cm x 2.2 cm x 0.45 cm) previously validated for use in older adult samples [24, 25]. The SENS device samples data at a rate of 12.5 Hz and an algorithm categorises data into predefined activities: lying/sitting rest; lying/sitting movement; standing; walking; sporadic walking; cycling & similar of light, moderate, and vigorous intensity. Residents will be asked to continually wear the tracker if possible, or for a minimum of 16 hours per day to ensure sufficient data collection. The manufacturer recommends attaching the device to the lateral distal thigh with a medical adhesive patch approximately 10 cm above the lateral epicondyle.

For peer review only

Fibion Emfit

The sleep sensor is the Fibion Emfit sensor (Emfit Corp. Kuopio, Finland) [19]. The device is a thin, flexible under-mattress device (32 cm x 62 cm x 0.4 cm) comprised of charged polymer layers and air voids, the position of which changes with mechanical pressure to measure body movement [26, 27]. The ballistic forces generated collect activity and physiological data during sleep including sleep time, sleep classes, heart rate, breathing rate, movement and bed occupancy and exits [27].

Phase one

Dashboard integration

A protocol will be developed for extracting data from digital technologies (wearable activity trackers, and sleep tracking sensors) and resident clinical information and medication records into the TCC-ADEPT platform. The TCC-ADEPT platform will include a dashboard with longitudinal visualisations of changes in residents' health and wellbeing over time (i.e., physical activity, sleep quality, blood pressure etc) using data loaded from the digital technologies and clinical data (see Figure 1). Medicine use (including sedative load and

For peer review only

cumulative medicines risk calculator) and pathology records (e.g., renal function) will also be uploaded to the dashboard. The platform infrastructure will be hosted on the Microsoft Azure cloud service with built-in regulatory compliance for the Health Insurance Portability and Accountability Act (HIPAA), and the Australian Government Information Security Manual (ISM). The resulting dashboard will be operated by the on-site pharmacist in phase two (feasibility study).

FIGURE 1

For peer review only

Identification of triggers for review

To determine whether a resident has experienced medicine-induced harm, the on-site pharmacists will review data from the digital technologies, residents' clinical and medicine data presented in the TCC-ADEPT dashboard. Baseline data collected within the first two weeks of the study will serve as the reference point, and the dashboard will present longitudinal data and trends for each resident. To determine whether changes in health and function are medicine-induced, visualisations will be presented relative to each time point where medicines are added, ceased, or modified.

In-built data analytics running an automated rules-based algorithm will be used for detection of changes in physiological or other health parameters. If and when residents' health declines beyond a certain threshold compared to baseline levels, the system will automatically flag residents that will alert the pharmacist (see Figure 2).

FIGURE 2

The trigger threshold will be determined based on minimum clinically significant difference where known in the literature (see Table 2). Where minimum clinically significant

differences are not known, a change of 10% will be considered a trigger, or in consultation with our clinicians.

Table 2. List of triggers

	Trigger (change from participants baseline value)
**Medicine data**	Sedative load increase by ≥2 [13].<br>Anticholinergic load increases ≥2 [13].<br>Number of medicines contributing to each adverse drug reaction listed in the Veterans' MATES adverse event cumulative risk calculator ≥2 [28].
**Resident care record**	Record of common medicine-induced symptoms e.g., constipation, dry mouth, delirium.<br>Hospital discharge or emergency department admission in the past week.
**Activity tracker**	≥30 minutes in daily sedentary time [14, 29].
**Sleep sensor**	≥10% reduction in sleep quality; ≥1 hour change in sleep time [30, 31].
**Blood pressure**	≥10 mmHg change in systolic; ≥5 mmHg in diastolic blood pressure [32].
**Weight**	≥5% change in weight [33].
**Blood glucose level**	≥10% change.

For peer review only

Note. mmHg= millimetres of mercury.

Phase two

The pre-post feasibility study will be conducted over 12-weeks. The study will be reported according to the CONSORT checklist for pilot and feasibility trials [34]. The sleep sensor (Fibion Emfit) [19] will be installed in the rooms of all enrolled and consenting residents, and study staff will provide residents with an activity tracker (Fibion SENS) [18]. Two weeks of baseline data will be collected from these technologies prior to the start of the pharmacist service.

Pharmacist service

The on-site pharmacist will monitor the participants using the TCC-ADEPT dashboard which includes data from the digital technologies as well as daily uploaded clinical and medicine data. Where clinically significant changes are detected, the resident will be automatically flagged for the pharmacist to take appropriate action (e.g., continue monitoring, perform a review, or initiate multidisciplinary review).

Sample size

For peer review only

A sample size of 30 to 50 participants is recommended for pilot and feasibility studies [35]. The current study aims to recruit 30 participants from a 120-bed residential aged care facility.

Outcomes and analysis

Study outcomes will be measured at 4-, 8-, and 12-weeks post-baseline period. Health outcomes include number of medication adverse events and change in resident symptom score (ESAS); surrogate measure outcomes include change in physical activity and sleep; process measure outcomes include number of pharmacist recommendations provided, record of staff time spent during study implementation period, and proportion of all pharmacists' recommendations to GP's and aged care staff that are implemented.

Process evaluation to assess the feasibility of the pharmacist service

A mixed methods evaluation, applying the RE-AIM evaluation framework [36] will be performed to assess the feasibility of the pharmacist service. Descriptive statistics will be employed to report demographic characteristics and health information obtained from the Resident Care Assessment Record.

For peer review only

295 To determine *reach*, the number and proportion of eligible residents who were willing to participate in the study will be calculated. Information that will be collected include number of residents approached by the study staff or pharmacist and provided with the study information, number of residents who meet the inclusion criteria, number of residents who provided consent, and number of participants who received the intervention and completed the 12-week intervention.

301 To determine *effectiveness*, changes in resident symptom score pre- and post-intervention, as measured using the Edmonton Symptom Assessment System (ESAS), will be assessed. Changes in medication use will be assessed, including changes to sedative and anticholinergic load pre- and post-intervention. Changes in 24-hour movement behaviour, as measured using the activity tracker, will be assessed pre- and post-intervention. The number of adverse events including falls (both injurious and non-injurious), fractures, delirium, faecal impaction, hospitalisation or emergency department presentation due to adverse medication events, and medication incidents will be assessed. The number of adverse events experienced by residents within the 12-weeks pre-intervention versus number of adverse events experienced during the 12-week intervention period will be compared.

For peer review only

To assess service *adoption*, the number and proportion of aged care residents approached that participated in the study will be calculated.

To assess *implementation*, the number and types of pharmacist interventions or recommendations will be recorded and evaluated. In addition, the proportion of recommendations made by the pharmacist will be compared with the number of recommendations accepted, to determine the acceptability of the intervention by the GPs and aged care staff. Semi-structured interviews or focus groups will be conducted with the project pharmacist, GPs, aged care staff, residents and family members to understand their views of the intervention, including facilitators and barriers to intervention delivery. An analysis will be undertaken of the costs associated with implementing the ADEPT-assisted pharmacist service. The resource use associated with the implementation of the ADEPT pharmacist service, including staff (aged care and health professionals) time, equipment, and resources will be recorded by trial staff prospectively. Changes in pharmacist time spent reviewing residents will be measured using pharmacist's record of amount of time spent reviewing medications for residents, generating recommendations and communicating with staff, GPs and residents. Resources will be valued using appropriate unit costs drawn from organisational finance data, relevant published employment awards [37], and the Medicare Benefits Schedule and Pharmaceutical Benefits Scheme [38, 39]. Where valuations are

For peer review only

unable to be obtained from published data, we will value using market rates, using the average cost from three suppliers. Prices will be updated to the current year using the Consumer Price Index [40]. A budget impact analysis will be conducted, extrapolating the within-study assessment of costs to a wider population level.

To assess *maintenance*, interviews with senior management at the aged care facility will be conducted to understand intention to continue to implement the service post study funding, and whether the service aligns to the facility's mission or sustainability of business model. The capacity of the aged care organisation to implement the service (beyond the feasibility study) to identify and negate potential medicine-induced harms will be explored.

Informed consent

Prior to the conduct of any study procedures, study staff will provide residents or an authorised representative with the participant information sheet and consent form. The purpose of the study and all study procedures will be discussed with the resident or an authorised representative. Informed written consent will be obtained by the resident or authorised representative. Participants will be assured that participation is completely voluntary and that they can withdraw from participation at any time.

For peer review only

For peer review only - http://bmjopen.bmj.com/site/about/guidelines.xhtml

Data management

All identifying information will be kept in secure settings in the residential aged care facility, University of South Australia (QUMPRC) or University of New South Wales, accessible only by central study staff. Where necessary, electronic tablets used for data collection (under password protection), and hard copies of questionnaires, will be stored temporarily within the residential aged care facility. The electronic tablets and forms will be stored in locked cabinets within the facility (accessible only by study staff). Information containing identifiers will be removed at the end of data collection by the project lead. The QUMPRC physical and IT environment operates to in-confidence and protected levels according to Department of Defence security standards.

Discussion

Detecting and preventing medicine-related harms is crucial given its high prevalence in residential aged care and the associated hospital admission and health-care costs [2]. This study has the potential to improve detection of medicine related harms (including unmanaged mild harms), mitigate associated adverse medicine events, and supplement

For peer review only

current resources in residential aged care. The ability to actively monitor aged care residents at risk of adverse events due to their medicine use will enable better opportunities for preventative intervention. Findings from this study are likely to inform aged care policy and support more effective implementation of the proposed aged care pharmacist service.

Digital technologies are not commonly used to monitor the effects of medicines in clinical practice including in the residential aged care setting. In our trial [9, 10], we tested two activity trackers, the GENEActiv [41] and Activinsights activity trackers [42], to monitor 24-hour movement behaviour among aged care residents. The GENEActiv is a research-grade activity tracker that allows high-resolution data collection (i.e., large data storage required) for up to 30 days before requiring charging, and therefore considered not suitable for use in our feasibility study involving data collection over 12-weeks. The Activinsights is a health-professional grade activity tracker with a lifespan of up to 1 year, however, its use was not maintained in our previous trial due to technical issues unrelated to the participants [9]. Both activity trackers are worn on the wrists. We selected the Fibion SENS activity tracker in the current study due to its non-invasive nature, as it can be conveniently worn on various locations (e.g., placed in a pocket). The Fibion SENS activity tracker can collect data for up to 22 weeks without requiring a recharge, and this tracker has been used and validated in older hospitalised patients [25] and older adults with knee osteoarthritis [24]. The

For peer review only

Fibion Emfit sleep sensor has been shown to provide an acceptable alternative to actigraphy for estimating bed entry and exit, and sleep wake times [43] as well as estimating beat-to-beat heart rate during sleep [44]. We foresee challenges with using the Fibion SENS (i.e., residents forgetting to wear them, losing sensors), but the aged care staff and pharmacist will be trained and are available to assist residents with device use.

The current study will recruit from a single aged-care facility, so findings may not generalise across other facilities or to independent living communities. There is no control group in this feasibility study. All enrolled participants will receive the intervention, which means that it will be difficult to determine whether any changes in participants' outcomes are due to the pharmacist service. However, the aim of the study is determining the feasibility and acceptability of the proposed pharmacist service. If shown to be feasible, the outcomes of this study will be used to design a future randomised controlled trial to test the effectiveness of the service on a larger multi-site scale. The intervention will be delivered by a single on-site pharmacist. This study will provide information on the time, resources and support required by the pharmacist to deliver the intervention, but a limitation is that most residential aged care in Australia do not have on-site pharmacists who are already familiar with their residents and therefore the resources and training required may differ depending on the level of experience of the pharmacists.

For peer review only

Risks

The aim of the current study is to test the feasibility of using digital technologies to help pharmacists better monitor the effects of medicines that the residents are taking. There are no apparent risks to participants that would be expected as a result of the pharmacist service or the unintrusive activity and sleep monitors. It is anticipated that there may be direct benefits to the participants through a reduction in adverse events.

Ethics and dissemination

Ethical approval has been obtained from the University of South Australia Human Research Ethics Committee (HREC205098). The study will be conducted in accordance with principles of the Declaration of Helsinki [45] and the National Statement of Ethical Conduct in Human Research [46]. During the study, any amendment to the study protocol or related documents will be submitted to the ethics committees for approval prior to implementation.

A Clinical Study Report in accordance with relevant ICH guidelines will be prepared, summarising all study data and analysis. Study findings may also be published or presented at scientific meetings. All publications arising from the study will comply with recognised

For peer review only

ethical standards concerning publications and authorships. In all instances, dissemination of the study results will include de-identified or average study data and will not identify an individual participant.

Data availability

Individual participant data will not be made available due to data being collected from a small group of aged care residents located at one facility in South Australia, and the high risk of re-identification of individual participants.

Trial Registration number

ACTRN12623000506695

Authors' Contributions

PB, TM, DD, RM, EER and RL conceived the study and obtained study funding. MSB, PB, JS, DD, TM and RL assisted in the development of the study protocol. MSB drafted the protocol manuscript and co-ordinates the day-to-day operation of the study. PB developed

For peer review only

the TeleClinical Care dashboard for the ADEPT study based on designs and prior development by NHL and PB. All authors critically revised and approved the final manuscript.

Funding statement

This project is funded by an Aged Care Research & Industry Innovation Australia (ARIIA) grant (GO00039). JS is employed by the organisation providing aged care service to the study participants. DD is supported by a Discovery Early Career Research Award (DE230101174). RL is supported by National Health and Medical Research Council Early Career Fellowship (APP1156368).

Competing interests statement

None declared.

Patient consent for publication

Not required.

For peer review only

References

1. Sluggett, J.K., et al., *Medication management policy, practice and research in Australian residential aged care: Current and future directions.* Pharmacological Research, 2017. **116**: p. 20-28.
2. Lim, R., et al., *The extent of medication-related hospital admissions in Australia: A review from 1988 to 2021.* Drug Safety, 2022. **45**(3): p. 249-257.
3. Handler, S.M., et al., *Epidemiology of medication-related adverse events in nursing homes.* The American Journal of Geriatric Pharmacotherapy, 2006. **4**(3): p. 264-272.
4. Al-Jumaili, A.A. and W.R. Doucette, *A systems approach to identify factors influencing adverse drug events in nursing homes.* Journal of the American Geriatrics Society, 2018. **66**(7): p. 1420-1427.
5. Tenhunen, M.L., B. Smithers, and B. Tucker, *Identifying medication-related adverse drug events in nursing facilities in east Texas.* The Consultant Pharmacist, 2016. **31**(8): p. 436-439.
6. Kalisch Ellett, L.M., et al., *Prevalence and Preventability of Adverse Medicine Events in a Sample of Australian Aged-Care Residents: A Secondary Analysis of Data from the ReMInDAR Trial.* Drug Safety, 2023. **46**(5): p. 493-500.

For peer review only

7. Sluggett, J.K., et al., *Provision of a comprehensive medicines review is associated with lower mortality risk for residents of aged care facilities: a retrospective cohort study.* Age and Ageing, 2022. **51**(7).

8. Australian Government Department of Health. *Aged care on-site pharmacist*. 2023; Available from: https://www.health.gov.au/our-work/aged-care-on-site-pharmacist.

9. Roughead, E.E., et al., *Effect of an ongoing pharmacist service to reduce medicine-induced deterioration and adverse reactions in aged-care facilities (nursing homes): a multicentre, randomised controlled trial (the ReMInDAR trial).* Age and Ageing, 2022. **51**(4).

10. Lim, R., et al., *Reducing medicine-induced deterioration and adverse reactions (ReMInDAR) trial: study protocol for a randomised controlled trial in residential aged-care facilities assessing frailty as the primary outcome.* 2020. **10**(4): p. e032851.

11. Lim, R., et al., *Pharmacists as patient advocates: A series of case studies illustrating the impacts of a regular pharmacist service in residential aged care (nursing homes).* Exploratory Research in Clinical and Social Pharmacy, 2023. **10**: p. 100268.

12. Pratt, N.L., et al., *Association between use of multiple psychoactive medicines and hospitalization for falls: retrospective analysis of a large healthcare claim database.* Drug Safety, 2014. **37**(7): p. 529-35.

For peer review only

13. Lim, R., et al., *Using wrist-worn accelerometers to identify the impact of medicines with anticholinergic or sedative properties on sedentary time: A 12-month prospective analysis.* Maturitas, 2023. **172**: p. 9-14.

14. Theou, O., et al., *Association between sedentary time and mortality across levels of frailty.* Canadian Medical Associtiion Journal, 2017. **189**(33): p. E1056-e1064.

15. Indraratna, P., et al., *Trials and tribulations: mHealth clinical trials in the COVID-19 pandemic.* Yearbook of Medical Informatics, 2021. **30**(1): p. 272-279.

16. Indraratna, P., et al., *A smartphone-based model of care to support patients with cardiac disease transitioning from hospital to the community (TeleClinical Care): Pilot randomized controlled trial.* Journal of Medical Internet Research, 2022. **10**(2): p. e32554.

17. Hui, D. and E. Bruera, *The Edmonton Symptom Assessment System 25 years later: Past, present, and future developments.* Journal of Pain Symptom Management, 2017. **53**(3): p. 630-643.

18. Fibion. *Fibion SENS*. 2020; Available from: https://sens.fibion.com/.

19. Fibion. *Fibion Emfit*. 2022; Available from: https://emfit.fibion.com/.

20. Roughead, E., et al., *Reducing harms from medicines in aged-care: findings from the ReMInDAR trial.* 2021, The University of South Australia.

For peer review only

21. Leecare, *P6Med*. 2023.

22. Medi-Map Group Pty Ltd, *Medi-Map*. 2023.

23. Nasreddine, Z.S., et al., *The Montreal Cognitive Assessment, MoCA: A brief screening tool for mild cognitive impairment.* Journal of the American Geriatrics Society, 2005. **53**(4): p. 695-699.

24. Ghaffari, A., et al., *Criterion calidity of linear accelerations measured with low-sampling-frequency accelerometers during overground walking in elderly patients with knee osteoarthritis.* Sensors, 2022. **22**(14): p. 5289.

25. Pedersen, B.S., et al., *Validation of two activity monitors in slow and fast walking hospitalized patients.* Rehabilitation Research and Practice, 2022. **2022**: p. 9230081.

26. Zink, M.D., et al., *Unobtrusive nocturnal heartbeat monitoring by a ballistocardiographic sensor in patients with sleep disordered breathing.* Scientific Reports, 2017. **7**(1): p. 13175.

27. Buimer, H.P., et al., *Sleep–wake monitoring of people with intellectual disability: Examining the agreement of EMFIT QS and actigraphy.* Journal of Applied Research in Intellectual Disabilities, 2023.

28. Veterans' MATES. *Cumulative risk calculator*. 2022; Available from: https://www.veteransmates.net.au/cumulative-risk-calculator.

For peer review only

29. Mankowski, R.T., et al., *Device-measured physical activity as a predictor of disability in mobility-limited older adults.* Journal of the American Geriatrics Society, 2017. **65**(10): p. 2251-2256.

30. Vincent, B.M., et al., *Sleeping time is associated with functional limitations in a national sample of older Americans.* Aging Clinical and Experimental Research, 2021. **33**(1): p. 175-182.

31. Goldman, S.E., et al., *Poor sleep is associated with poorer physical performance and greater functional limitations in older women.* Sleep, 2007. **30**(10): p. 1317-24.

32. Guzman, H., et al., *Clinically significant blood pressure reduction among participants of a lifestyle intervention improvement program strongly associated with reduced systemic inflammation in a high risk working population.* Circulation, 2014. **129**(suppl_1): p. AP391-AP391.

33. Williamson, D.A., G.A. Bray, and D.H. Ryan, *Is 5% weight loss a satisfactory criterion to define clinically significant weight loss?* Obesity 2015. **23**(12): p. 2319-20.

34. Eldridge, S.M., et al., *CONSORT 2010 statement: extension to randomised pilot and feasibility trials.* BMJ, 2016. **355**: p. i5239.

For peer review only

35. Sim, J. and M. Lewis, *The size of a pilot study for a clinical trial should be calculated in relation to considerations of precision and efficiency.* Journal of Clinical Epidemiology, 2012. **65**(3): p. 301-8.

36. Glasgow, R.E., et al., *RE-AIM planning and evaluation framework: Adapting to new science and practice with a 20-year review.* Frontiers in Public Health, 2019. **7**: p. 64.

37. Australian Government Fair Work Ombudsman. *Fair Work Ombudsman*. Available from: https://www.fairwork.gov.au/.

38. Australian Government Department of Health and Aged Care. *MBS Online contains the Medicare Benefits Schedule (MBS), a listing of the Medicare services subsidised by the Australian Government*. 2023; Available from: http://www.mbsonline.gov.au/internet/mbsonline/publishing.nsf/Content/Home.

39. Australian Government Department of health and Aged Care. *Pharmaceutical Benefits Scheme (PBS)*. 2023; Available from: https://www.pbs.gov.au/pbs/home.

40. Australian Bureau of Statistics. *Price indexes and inflation*. 2023.

41. Esliger, D.W., et al., *Validation of the GENEA accelerometer.* Medicine and Science in Sports and Exercise, 2011. **43**(6): p. 1085-93.

42. ActivInsights Ltd. *Professional wearables*. 2017.

For peer review only

553 43. Piantino, J., et al., *Emfit bed sensor activity shows strong agreement with wrist*
554 *actigraphy for the assessment of sleep in the home setting.* Nature and Science of
555 Sleep, 2021. **13**: p. 1157-1166.

556 44. Antink, C.H., et al., *Ballistocardiography can estimate beat-to-beat heart rate*
557 *accurately at night in patients after vascular intervention.* IEEE Journal of Biomedical
558 and Health Informatics, 2020. **24**(8): p. 2230-2237.

559 45. World Medical Association, *World medical association declaration of Helsinki: Ethical*
560 *principles for medical research involving human subjects.* JAMA, 2013. **310**(20): p.
561 2191-2194.

562 46. National Health and Medical Research Council. *National Statement of Ethical*
563 *Conduct in Human Research.* 2007; Available from: https://www.nhmrc.gov.au/about-
564 us/publications/national-statement-ethical-conduct-human-research-2007-updated-
565 2018.

566

567

568

569

For peer review only

Figure 1A shows example vital signs (blood pressure, body weight, and oxygen saturation) with onset of a new medication (Alprazolam 0.25 mg tds) overlaid in the TCC-ADEPT platform. Figure 1B shows example cumulative risks and type of adverse drug reactions displayed in the TCC-ADEPT platform.

Figure 2. Proposed service flow

For peer review only

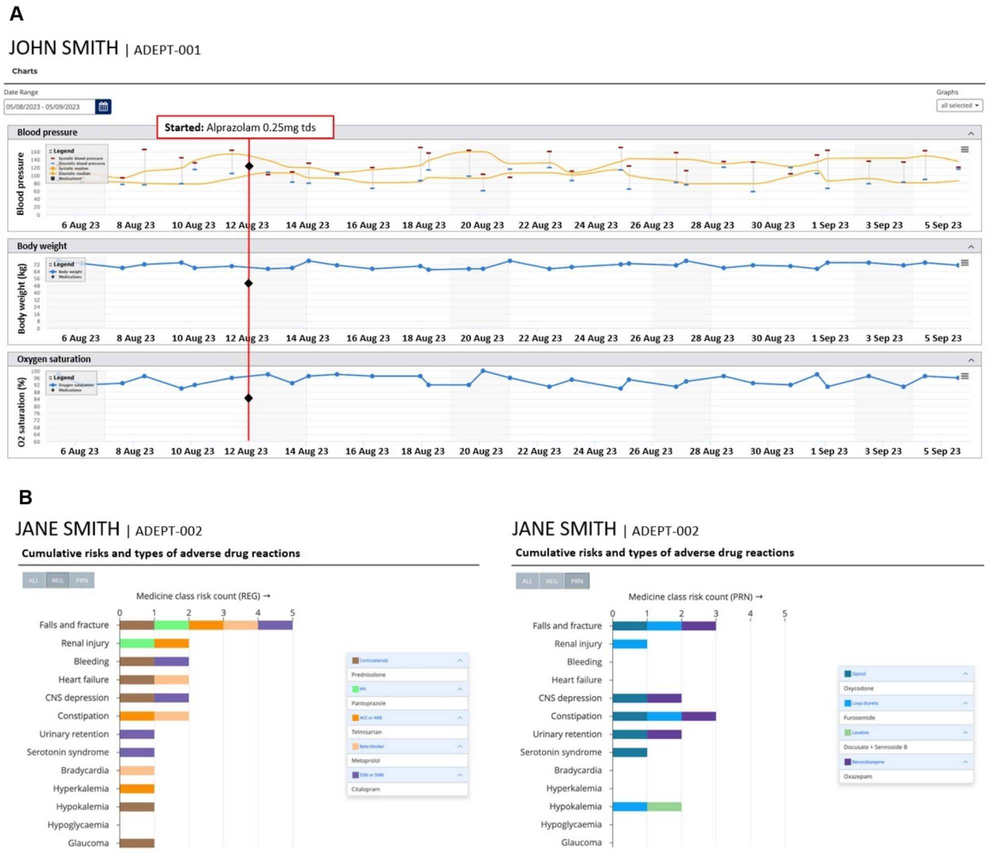

Figure 1A shows example vital signs (blood pressure, body weight, and oxygen saturation) with onset of a new medication (Alprazolam 0.25 mg tds) overlaid in the TCC-ADEPT platform. Figure 1B shows example cumulative risks and type of adverse drug reactions displayed in the TCC-ADEPT platform.

223x194mm (144 x 144 DPI)

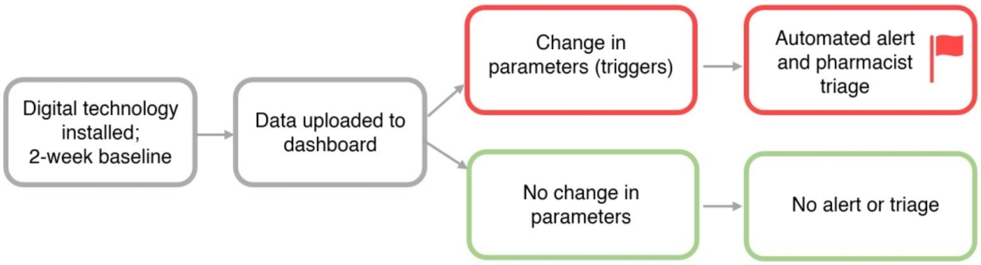

Figure 2. Proposed service flow

277x81mm (144 x 144 DPI)

SPIRIT

STANDARD PROTOCOL ITEMS: RECOMMENDATIONS FOR INTERVENTIONAL TRIALS

SPIRIT 2013 Checklist: Recommended items to address in a clinical trial protocol and related documents*

Section/item	Item No	Description	Addressed on page number
**Administrative information**			
Title	1	Descriptive title identifying the study design, population, interventions, and, if applicable, trial acronym	______1______
Trial registration	2a	Trial identifier and registry name. If not yet registered, name of intended registry	______3______
	2b	All items from the World Health Organization Trial Registration Data Set	_____N/A_____
Protocol version	3	Date and version identifier	_____N/A_____
Funding	4	Sources and types of financial, material, and other support	_____21______
Roles and responsibilities	5a	Names, affiliations, and roles of protocol contributors	____1 & 20____
	5b	Name and contact information for the trial sponsor	_____21______
	5c	Role of study sponsor and funders, if any, in study design; collection, management, analysis, and interpretation of data; writing of the report; and the decision to submit the report for publication, including whether they will have ultimate authority over any of these activities	_____N/A_____
	5d	Composition, roles, and responsibilities of the coordinating centre, steering committee, endpoint adjudication committee, data management team, and other individuals or groups overseeing the trial, if applicable (see Item 21a for data monitoring committee)	_____N/A_____

For peer review only - http://bmjopen.bmj.com/site/about/guidelines.xhtml

Introduction

Background and rationale	6a	Description of research question and justification for undertaking the trial, including summary of relevant studies (published and unpublished) examining benefits and harms for each intervention	____4 & 5_____
	6b	Explanation for choice of comparators	______19_____
Objectives	7	Specific objectives or hypotheses	____5 & 6_____
Trial design	8	Description of trial design including type of trial (eg, parallel group, crossover, factorial, single group), allocation ratio, and framework (eg, superiority, equivalence, noninferiority, exploratory)	______6______

Methods: Participants, interventions, and outcomes

Study setting	9	Description of study settings (eg, community clinic, academic hospital) and list of countries where data will be collected. Reference to where list of study sites can be obtained	______7______
Eligibility criteria	10	Inclusion and exclusion criteria for participants. If applicable, eligibility criteria for study centres and individuals who will perform the interventions (eg, surgeons, psychotherapists)	______7______
Interventions	11a	Interventions for each group with sufficient detail to allow replication, including how and when they will be administered	___11 – 14____
	11b	Criteria for discontinuing or modifying allocated interventions for a given trial participant (eg, drug dose change in response to harms, participant request, or improving/worsening disease)	_____17______
	11c	Strategies to improve adherence to intervention protocols, and any procedures for monitoring adherence (eg, drug tablet return, laboratory tests)	____18 & 19____
	11d	Relevant concomitant care and interventions that are permitted or prohibited during the trial	______7______
Outcomes	12	Primary, secondary, and other outcomes, including the specific measurement variable (eg, systolic blood pressure), analysis metric (eg, change from baseline, final value, time to event), method of aggregation (eg, median, proportion), and time point for each outcome. Explanation of the clinical relevance of chosen efficacy and harm outcomes is strongly recommended	___15 – 17____
Participant timeline	13	Time schedule of enrolment, interventions (including any run-ins and washouts), assessments, and visits for participants. A schematic diagram is highly recommended (see Figure)	____7 – 10____

For peer review only - http://bmjopen.bmj.com/site/about/guidelines.xhtml

Sample size	14	Estimated number of participants needed to achieve study objectives and how it was determined, including clinical and statistical assumptions supporting any sample size calculations	_____15______
Recruitment	15	Strategies for achieving adequate participant enrolment to reach target sample size	____7 & 15_____

Methods: Assignment of interventions (for controlled trials)

Allocation:

Sequence generation	16a	Method of generating the allocation sequence (eg, computer-generated random numbers), and list of any factors for stratification. To reduce predictability of a random sequence, details of any planned restriction (eg, blocking) should be provided in a separate document that is unavailable to those who enrol participants or assign interventions	_____N/A_____
Allocation concealment mechanism	16b	Mechanism of implementing the allocation sequence (eg, central telephone; sequentially numbered, opaque, sealed envelopes), describing any steps to conceal the sequence until interventions are assigned	_____N/A_____
Implementation	16c	Who will generate the allocation sequence, who will enrol participants, and who will assign participants to interventions	_____N/A_____
Blinding (masking)	17a	Who will be blinded after assignment to interventions (eg, trial participants, care providers, outcome assessors, data analysts), and how	_____N/A_____
	17b	If blinded, circumstances under which unblinding is permissible, and procedure for revealing a participant's allocated intervention during the trial	_____N/A_____

Methods: Data collection, management, and analysis

Data collection methods	18a	Plans for assessment and collection of outcome, baseline, and other trial data, including any related processes to promote data quality (eg, duplicate measurements, training of assessors) and a description of study instruments (eg, questionnaires, laboratory tests) along with their reliability and validity, if known. Reference to where data collection forms can be found, if not in the protocol	___7 – 11_____
	18b	Plans to promote participant retention and complete follow-up, including list of any outcome data to be collected for participants who discontinue or deviate from intervention protocols	_____N/A_____

Data management	19	Plans for data entry, coding, security, and storage, including any related processes to promote data quality (eg, double data entry; range checks for data values). Reference to where details of data management procedures can be found, if not in the protocol	___11 & 17____
Statistical methods	20a	Statistical methods for analysing primary and secondary outcomes. Reference to where other details of the statistical analysis plan can be found, if not in the protocol	___15 – 17____
	20b	Methods for any additional analyses (eg, subgroup and adjusted analyses)	_____N/A_____
	20c	Definition of analysis population relating to protocol non-adherence (eg, as randomised analysis), and any statistical methods to handle missing data (eg, multiple imputation)	_____N/A_____
**Methods: Monitoring**			
Data monitoring	21a	Composition of data monitoring committee (DMC); summary of its role and reporting structure; statement of whether it is independent from the sponsor and competing interests; and reference to where further details about its charter can be found, if not in the protocol. Alternatively, an explanation of why a DMC is not needed	_____N/A_____
	21b	Description of any interim analyses and stopping guidelines, including who will have access to these interim results and make the final decision to terminate the trial	_____N/A_____
Harms	22	Plans for collecting, assessing, reporting, and managing solicited and spontaneously reported adverse events and other unintended effects of trial interventions or trial conduct	_____19______
Auditing	23	Frequency and procedures for auditing trial conduct, if any, and whether the process will be independent from investigators and the sponsor	_____N/A_____
**Ethics and dissemination**			
Research ethics approval	24	Plans for seeking research ethics committee/institutional review board (REC/IRB) approval	_____19______
Protocol amendments	25	Plans for communicating important protocol modifications (eg, changes to eligibility criteria, outcomes, analyses) to relevant parties (eg, investigators, REC/IRBs, trial participants, trial registries, journals, regulators)	___19 – 20____

Consent or assent	26a	Who will obtain informed consent or assent from potential trial participants or authorised surrogates, and how (see Item 32)	______17_______
	26b	Additional consent provisions for collection and use of participant data and biological specimens in ancillary studies, if applicable	______N/A______
Confidentiality	27	How personal information about potential and enrolled participants will be collected, shared, and maintained in order to protect confidentiality before, during, and after the trial	_____17 & 20_____
Declaration of interests	28	Financial and other competing interests for principal investigators for the overall trial and each study site	______21_______
Access to data	29	Statement of who will have access to the final trial dataset, and disclosure of contractual agreements that limit such access for investigators	______17_______
Ancillary and post-trial care	30	Provisions, if any, for ancillary and post-trial care, and for compensation to those who suffer harm from trial participation	______N/A______
Dissemination policy	31a	Plans for investigators and sponsor to communicate trial results to participants, healthcare professionals, the public, and other relevant groups (eg, via publication, reporting in results databases, or other data sharing arrangements), including any publication restrictions	______20_______
	31b	Authorship eligibility guidelines and any intended use of professional writers	______20_______
	31c	Plans, if any, for granting public access to the full protocol, participant-level dataset, and statistical code	______20_______
**Appendices**			
Informed consent materials	32	Model consent form and other related documentation given to participants and authorised surrogates	______N/A______
Biological specimens	33	Plans for collection, laboratory evaluation, and storage of biological specimens for genetic or molecular analysis in the current trial and for future use in ancillary studies, if applicable	______N/A______

*It is strongly recommended that this checklist be read in conjunction with the SPIRIT 2013 Explanation & Elaboration for important clarification on the items. Amendments to the protocol should be tracked and dated. The SPIRIT checklist is copyrighted by the SPIRIT Group under the Creative Commons "Attribution-NonCommercial-NoDerivs 3.0 Unported" license.

BMJ Open

A Digitally Enabled, Pharmacist service to detecT medicine harms in residential aged care (nursing home) (ADEPT): protocol for a feasibility study.

Journal:	*BMJ Open*
Manuscript ID	bmjopen-2023-080148.R1
Article Type:	Protocol
Date Submitted by the Author:	23-Jan-2024
Complete List of Authors:	Boord, Monique; University of South Australia, Quality Use of Medicines and Pharmacy Research Centre<br>Brown, Peter; University of New South Wales, Tyree Foundation Institute of Health Engineering (IHealthE) and Graduate School of Biomedical Engineering<br>Soriano, Julian; Tanunda Lutheran Home Inc; SA Pharmacy<br>Meola, Tahlia; University of South Australia, UniSA Clinical and Health Sciences<br>Dumuid, Dorothea ; University of South Australia, Alliance for Research in Exercise, Nutrition and Activity<br>Milte, Rachel; Flinders University, Rehabilitation, Aged and Extended Care<br>Roughead, Elizabeth; University of South Australia, Quality Use of Medicines and Pharmacy Research Centre, UniSA Clinical and Health Sciences<br>Lovell, Nigel; University of New South Wales, Tyree Foundation Institute of Health Engineering (IHealthE) and Graduate School of Biomedical Engineering<br>Stone, Helen; Pharmaceutical Society of Australia<br>Whitehouse, Joseph; HealthyCare Services<br>Janetzki, Jack; University of South Australia, Quality Use of Medicines and Pharmacy Research Centre; University of South Australia, UniSA Clinical and Health Sciences<br>Gebreyohannes, Eyob Alemayehu; University of South Australia, Quality Use of Medicines and Pharmacy Research Centre, UniSA Clinical and Health Sciences; The University of Western Australia, School of Allied Health<br>Lim, Renly; University of South Australia, Quality Use of Medicines and Pharmacy Research Centre, UniSA Clinical and Health Sciences
**Primary Subject Heading**:	Geriatric medicine
Secondary Subject Heading:	Health services research
Keywords:	Adverse events < THERAPEUTICS, Aged, Health Services for the Aged, GERIATRIC MEDICINE, Health policy < HEALTH SERVICES ADMINISTRATION & MANAGEMENT, Patient-Centered Care

SCHOLARONE™
Manuscripts

A Digitally Enabled, Pharmacist service to detecT medicine harms in residential aged care (nursing home) (ADEPT): protocol for a feasibility study.

Authors

Monique S Boord[1], Peter Brown[2], Julian Soriano[3,4], Tahlia Meola[5], Dorothea Dumid[6], Rachel Milte[7], Elizabeth E Roughead[1], Nigel H. Lovell[2], Helen Stone[8], Joseph Whitehouse[9], Jack L Janetzki[1,5], Eyob Alemayehu Gebreyohannes[1,10], Renly Lim[1]

Institutions

[1] Quality Use of Medicines and Pharmacy Research Centre, UniSA Clinical and Health Sciences, University of South Australia, Adelaide, Australia

[2] Tyree Foundation Institute of Health Engineering (IHealthE) and Graduate School of Biomedical Engineering, UNSW, Sydney, Australia

[3] Tanunda Lutheran Home, Tanunda, Australia

[4] SA Pharmacy, Adelaide, Australia

[5] UniSA Clinical and Health Sciences, University of South Australia, Adelaide, Australia

[6] Alliance for Research in Exercise, Nutrition and Activity, UniSA Clinical and Health Sciences, University of South Australia, Adelaide, Australia

For peer review only

[7] Caring Futures Institute, College of Nursing and Health Sciences, Flinders University, Adelaide, Australia

[8] Pharmaceutical Society of Australia, Deakin, Australia

[9] HealthyCare Services, Welland, Australia

[10] School of Allied Health, The University of Western Australia, Perth, Australia

Correspondence to:

Dr Monique Boord

Quality Use of Medicines and Pharmacy Research Centre,

UniSA Clinical and Health Sciences,

University of South Australia, Adelaide, Australia

Monique.boord@unisa.edu.au

Abstract

Introduction

This feasibility study aims to develop and test a new model of practice in Australia: using digital technologies to enable pharmacists to monitor early signs and symptoms of medicine-induced harms in residential aged care.

For peer review only

Methods and analysis

Thirty residents will be recruited from an aged care facility in South Australia. The study will be conducted in two phases. In phase 1, the study team will work with aged care software providers and developers of digital technologies (a wearable activity tracker and a sleep tracking sensor) to gather physical activity and sleep data, as well as medication and clinical data from the electronic medication management system and aged care clinical software. Data will be centralised into a cloud-based monitoring platform (TeleClinical Care). The TeleClinical Care (TCC) will be used to create dashboards that will include longitudinal visualisations of changes in residents' health, function, and medicine use over time. In Phase 2, the on-site pharmacist will use the centralised TCC platform to monitor each resident's medicine, clinical, physical activity and sleep data to identify signs of medicine-induced harms over a 12-week period.

A mixed methods process evaluation applying the RE-AIM evaluation framework will be used to assess the feasibility of the service. Outcome measures include service reach, changes in resident symptom scores (measured using the Edmonton Symptom Assessment System), number of medication adverse events detected, changes in physical activity and

For peer review only

sleep, number of pharmacist recommendations provided, cost analysis, and proportion of all pharmacists' recommendations implemented at 4-, 8-, and 12-weeks post-baseline period.

Ethics and dissemination

Ethical approval has been obtained from the University of South Australia's Human Research Ethics Committee (205098). Findings will be disseminated through published manuscripts, conference presentations, and reporting to the study funder.

Trial registration number

ACTRN12623000506695

Strengths and limitations of this study

- This study is the first to develop and implement a centralised digital platform in residential aged care to enable pharmacists to actively monitor medicine-induced harms.
- Uses objective assessments of changes in physical activity and sleep to identify "mild" medicine harms.

For peer review only

- Due to recruiting from a single residential aged care facility in South Australia, findings may not generalise across all residents living in aged care.
- Lack of compliance with use and wearing of digital technologies by residents is a potential limitation.

For peer review only

Introduction

Approximately eight percent of adults in Australia aged 65 and older reside in aged-care facilities (nursing home) [1], with up to 63% receiving nine or more regular medications [1]. Medicine-related problems contribute to an estimated 250,000 hospital admissions in Australia every year, with an estimated annual cost of $1.4 billion [2]. Medicine-related harms are of significant concern in residential aged care, with up to 27 adverse medicine events occurring per 100 residents per month [3-5]. In Australia, about one in five persons living in residential aged care experience an adverse medicine event every month; of which 83% were potentially preventable [6]. Despite the significant medicine-related harms, most Australian residential aged care facilities have limited capacity to provide clinical pharmacy services; existing services are limited to government funded residential medication management review (comprehensive medication reviews performed by accredited pharmacists in residential aged care) and quality use of medicine services [7].

The Australian Government, recognising the significance of medicine-related problems in aged care, committed $350 million to improve medication safety in residential aged care through funding for pharmacists [8]. The government investment presents new opportunities for pharmacists to review residents for signs and symptoms of medicine harms more frequently. The present study builds on our recent randomised controlled trial which

For peer review only

investigated the effectiveness of ongoing pharmacist assessments compared with usual care in reducing medicine induced deterioration, frailty, and adverse events across 39 residential aged care facilities in Australia [9, 10]. Our study showed that one in five residents experienced a preventable adverse event (e.g., falls, fractures) every month due to medication use [9]. The pharmacists identified and resolved medicine-related harms and served as an advocate for residents. Further, significant differences in change in cognition from baseline were observed between intervention and control groups [9]. The service ensured pharmacist engagement with residents, rather than just reviewing medicines, which enabled the advocacy [11].

Frequent monitoring of medicine-induced harms is important because medicines can cause mild harm (e.g., sedation, poor balance) that often remains undetected, but can lead to serious adverse events in older people if left unmanaged. For example, commonly prescribed psychotropic medicines can cause sedation and poor cognition that may lead to injurious falls and fractures [12]. An increase of sedative load from two to four over a 12-month period has been associated with an average increase in daily sedentary behaviour of approximately 24 minutes [13]; an increase of 60 minutes per day of sedentary time is associated with a 19% increase in risk of death [14]. Detecting mild, cumulative effects of

For peer review only

medicine-induced harms, including changes in physical activity, is likely to be important, however, this can be difficult and labour intensive to measure and monitor.

Harnessing digital technologies to enable active monitoring of medicine-related harms is one potential strategy to enabling proactive monitoring. For pharmacists to effectively monitor harms, they will not only need to review medicines, but also recognise and monitor early signs of medicine-induced harms such as changes in sleep, activity, or cognition [9, 13]. Digital technologies can measure changes in sleep, activity, or cognition objectively, which may enhance effective communication about medicines-related harm across aged care residents' care teams, including to general practitioners (GPs), nurses and care staff, as well as support the recommendations from pharmacists and their acceptance by the team and residents themselves. The TeleClinical-Care (TCC) platform has been used successfully during the COVID-19 pandemic to safely manage more than 8,000 COVID-positive patients as they self-isolated at home [15] and is being used in trials to manage cardiovascular disease and stroke [16].

The ADEPT project aims to develop and implement a digitally enabled, evidence-based, pharmacist service built upon TCC (TCC-ADEPT) to actively detect medicine harms in residential aged care. Specifically, we will:

For peer review only

1. Determine the number and proportion of eligible residents who were willing to participate in the study;
2. Determine changes in resident outcomes, including changes in symptoms as measured using the Edmonton Symptom Assessment System (ESAS) [17], physical activity and sleep (24-hour movement behaviour), and adverse events including falls (both injurious and non-injurious), fractures, delirium, faecal impaction, hospitalisation or emergency department presentation due to adverse medication events, and medication incidents, pre- and post-digital pharmacist service;
3. Determine the number and types of pharmacist recommendations that resulted following use of the TCC-ADEPT platform, and the number of pharmacist recommendations that were accepted and implemented by GPs and aged care staff;
4. Explore the acceptability of the digital pharmacist service by residents and family members, aged care staff and GPs;
5. Assess the costs associated with implementing the TCC-ADEPT platform and potential cost impacts to the health and aged care system in wider-spread roll out of the platform;

For peer review only

6. Explore the residential aged care facility's intention to continue to implement the pharmacist service post study funding and whether the service aligns to the facility's mission or sustainability of business model.

We hypothesise that the new pharmacist service is feasible and acceptable to aged care staff, residents and their GPs, and can enhance detection of early signs and symptoms of medicine harms, triggering review and action when required.

Methods and analysis

Study design

The ADEPT project will be conducted in two phases over a 12-month period. Phase one will include gathering data from digital technologies, medication and health records into the TCC platform. Digital technologies will include an activity tracker [18] and a sleep sensor [19]. A pre-post12-week implementation study will constitute phase two of the project. The on-site pharmacist will monitor all participants using the centralised TCC-ADEPT platform, using data to inform resident reviews and to initiate conversations with residents, GPs and aged care staff on medicine, health and quality of life changes for residents. Recruitment and study implementation will occur from August 2023 until February 2024.

For peer review only

Patient and public involvement

Aged care residents, representatives and health professionals were directly involved in the development or design of this study. Findings from interviews with residents, carers and aged care staff from the ReMInDAR trial were used to inform the development and design of this study [20].

Cohort selection

Residents will be considered eligible to participate if they are a permanent resident living in the designated residential aged care facility. Exclusion criteria include receiving palliative or respite care.

Screening and recruitment

Residents will be recruited from an aged care facility in South Australia. Promotional recruitment material (flyers) will be distributed onsite in common areas of the facility one month prior to study commencement. Participant information sheets will be provided to residents deemed potentially eligible by facility staff, and will be given to all residents willing to participate, informing them of the study details and pharmacist service.

Study staff will perform initial screening of residents' care record to identify potentially eligible residents. Screening logs will be kept monitoring residents' recruitment and to enable

For peer review only

a description of the study population from which eligible residents have been enrolled. If the resident fulfills inclusion criteria based on the initial screening process, the on-site pharmacist will invite the resident to participate in the study and written informed consent will be obtained from the resident or their authorised representative (model consent form shown in Supplementary Material).

Baseline data collection

Once written consent is obtained, study staff will collect baseline data from the Resident Care Assessment Record held electronically in Leecare [21] and Medi-Map [22] software, which contains demographic details, comorbidities, medicine use, falls history, and care provided within the aged care facility. Study staff will administer the Edmonton Symptom Assessment Scale (ESAS) [17] and the Montreal Cognitive Assessment (MoCA) [23] at baseline. An observational logbook or diary will be kept to record information not collected in the existing data fields. Table 1 indicates data collected at each data collection time points.

For peer review only

Table 1. Data collection schedule

Variable	Description	Data source	Data collection time points			
			Baseline	4-weeks	8-weeks	12-weeks
**Demographic**	Age, gender	Leecare	✓			
**Medical conditions**	Diagnosis of medical condition	Leecare	✓	✓	✓	✓
**Medications***	Name, strength, dose, frequency of medicine administration	Medi-Map	✓	✓	✓	✓
**Nutritional status**	Swallowing status	Leecare	✓			✓
**Functional ability**	Mobilisation status	Leecare	✓			✓
**Cognitive function**	Montreal Cognitive Assessment	Leecare	✓			✓
**Falls history**	Number of falls and related health outcomes	Leecare	✓			✓
**Vital signs**	Height, weight, blood pressure (systolic and diastolic), respirations, pulse, BGL, SpO2	Leecare	✓	✓	✓	✓
**Pain**	PainChek® score severity across 6 domains	Leecare	✓	✓	✓	✓
**Residential Medication Management Review (RMMR)**	Dates of RMMR provision will be recorded	On-site aged care pharmacist	✓	✓	✓	✓
**Physical activity**	Time spent in light, moderate, vigorous-intensity activity	Fibion SENS device	✓	✓	✓	✓
**Sleep**	Movements during sleep, durations of light, deep and REM sleep	Fibion Emfit device	✓	✓	✓	✓
**Edmonton Symptom Assessment Scale (ESAS)**	Severity across 10 symptoms	Participant or third-party informant review	✓	✓	✓	✓
**Adverse events**	E.g., falls, fracture, delirium, faecal impaction, incontinence	Leecare, Pharmacists,	✓	✓	✓	✓

For peer review only

		aged care staff, residents				
**Incidents**	Medication incidents will be recorded including date/time and severity	Leecare, Pharmacists, aged care staff, residents	✓	✓	✓	✓
**Ambulance call-outs**	Date and reasons will be recorded	Leecare, Pharmacists, aged care staff, residents	✓	✓	✓	✓
**Hospital visits**	Date and reason of emergency department visits or hospital admission will be recorded	Leecare, Pharmacists, aged care staff, residents	✓	✓	✓	✓
**All-cause mortality**	Any deaths in the 12-week study period will be recorded	Aged care staff		✓	✓	✓

Note* data will be collected and exported daily into the TCC-ADEPT platform over the 12-week study period. BGL= Blood glucose level; SpO2= oxygen saturation; REM= rapid eye movement.

Measures

Edmonton Symptom Assessment Scale (ESAS)

The ESAS is a 10-item numeric rating scale assessing self-reported pain, fatigue, nausea, depression, anxiety, drowsiness, shortness of breath, appetite, wellbeing, and sleep [17]. Participants or their carer will rate the severity of each symptom on a scale of 0-10 by circling a number on each scale which best describes their symptoms over the past 24 hours [17]. Higher scores indicate higher symptom burden.

For peer review only

Montreal Cognitive Assessment (MoCA)

The MoCA is a 1 page, 30-point screening instrument that assesses cognitive domains of memory, language, executive functions, visuospatial skills, calculation, abstraction, attention, concentration, and orientation [23]. A cut off score of 26 has been shown to differentiate mild cognitive impairment or dementia from normal cognition [23]. When scoring, a one-point adjustment is given to those with 12 or less years of formal education [23].

Fibion SENS

The activity tracker is the Fibion SENS device (SENS Motion®, Copenhagen, Denmark) [18] which is a waterproof 3D triaxial accelerometer (4.7 cm x 2.2 cm x 0.45 cm) previously validated for use in older adult samples [24, 25]. The SENS device samples data at a rate of 12.5 Hz and an algorithm categorises data into predefined activities: lying/sitting rest; lying/sitting movement; standing; walking; sporadic walking; cycling & similar of light, moderate, and vigorous intensity. Residents will be asked to continually wear the tracker if possible, or for a minimum of 16 hours per day to ensure sufficient data collection. The manufacturer recommends attaching the device to the lateral distal thigh with a medical adhesive patch approximately 10 cm above the lateral epicondyle.

For peer review only

Fibion Emfit

The sleep sensor is the Fibion Emfit sensor (Emfit Corp. Kuopio, Finland) [19]. The device is a thin, flexible under-mattress device (32 cm x 62 cm x 0.4 cm) comprised of charged polymer layers and air voids, the position of which changes with mechanical pressure to measure body movement [26, 27]. The ballistic forces generated collect activity and physiological data during sleep including sleep time, sleep classes, heart rate, breathing rate, movement and bed occupancy and exits [27].

Phase one

Dashboard integration

A protocol will be developed for extracting data from digital technologies (wearable activity trackers, and sleep tracking sensors) and resident clinical information and medication records into the TCC-ADEPT platform. The TCC-ADEPT platform will include a dashboard with longitudinal visualisations of changes in residents' health and wellbeing over time (i.e., physical activity, sleep quality, blood pressure etc) using data loaded from the digital technologies and clinical data (see Figure 1). Medicine use (including sedative load and

For peer review only

cumulative medicines risk calculator) and pathology records (e.g., renal function) will also be uploaded to the dashboard. The platform infrastructure will be hosted on the Microsoft Azure cloud service with built-in regulatory compliance for the Health Insurance Portability and Accountability Act (HIPAA), and the Australian Government Information Security Manual (ISM). The resulting dashboard will be operated by the on-site pharmacist in phase two (feasibility study).

FIGURE 1

For peer review only

Identification of triggers for review

To determine whether a resident has experienced medicine-induced harm, the on-site pharmacists will review data from the digital technologies, residents' clinical and medicine data presented in the TCC-ADEPT dashboard. Baseline data collected within the first two weeks of the study will be averaged and serve as the reference point, and the dashboard will present longitudinal data and trends for each resident. To determine whether changes in health and function are medicine-induced, visualisations will be presented relative to each time point where medicines are added, ceased, or modified.

In-built data analytics running an automated rules-based algorithm will be used for detection of changes in physiological or other health parameters. If and when residents' health declines beyond a certain threshold compared to baseline levels, the system will automatically flag residents that will alert the pharmacist (see Figure 2).

FIGURE 2

The trigger threshold will be determined based on minimum clinically significant difference where known in the literature (see Table 2). Where minimum clinically significant

For peer review only

differences are not known, a change of 10% will be considered a trigger, or in consultation with our clinicians.

Table 2. List of triggers

	Trigger (change from participants baseline value)
**Medicine data**	Sedative load increase by ≥2 [13].<br>Anticholinergic load increases ≥2 [13].<br>Number of medicines contributing to each adverse drug reaction listed in the Veterans' MATES adverse event cumulative risk calculator ≥2 [28].
**Resident care record**	Record of common medicine-induced symptoms e.g., constipation, dry mouth, delirium.<br>Hospital discharge or emergency department admission in the past week.
**Activity tracker**	≥30 minutes in daily sedentary time [14, 29].
**Sleep sensor**	≥10% reduction in sleep quality; ≥1 hour change in sleep time [30, 31].
**Blood pressure**	≥10 mmHg change in systolic; ≥5 mmHg in diastolic blood pressure [32].
**Weight**	≥5% change in weight [33].
**Blood glucose level**	≥10% change.

For peer review only

Note. mmHg= millimetres of mercury.

Phase two

The pre-post feasibility study will be conducted over 12-weeks. The study will be reported according to the CONSORT checklist for pilot and feasibility trials [34]. The sleep sensor (Fibion Emfit) [19] will be installed in the rooms of all enrolled and consenting residents, and study staff will provide residents with an activity tracker (Fibion SENS) [18]. Two weeks of baseline data will be collected from these technologies prior to the start of the pharmacist service.

Pharmacist service

The on-site pharmacist will monitor the participants using the TCC-ADEPT dashboard which includes data from the digital technologies as well as daily uploaded clinical and medicine data. Where clinically significant changes are detected, the resident will be automatically flagged for the pharmacist to take appropriate action (e.g., continue monitoring, perform a review, or initiate multidisciplinary review).

Sample size

For peer review only

A sample size of 30 to 50 participants is recommended for pilot and feasibility studies [35]. The current study aims to recruit 30 participants from a 120-bed residential aged care facility.

Outcomes and analysis

Study outcomes will be measured at 4-, 8-, and 12-weeks post-baseline period. Health outcomes include number of medication adverse events and change in resident symptom score (ESAS); surrogate measure outcomes include change in physical activity and sleep; process measure outcomes include number of pharmacist recommendations provided, record of staff time spent during study implementation period, and proportion of all pharmacists' recommendations to GP's and aged care staff that are implemented.

Process evaluation to assess the feasibility of the pharmacist service

A mixed methods evaluation, applying the RE-AIM evaluation framework [36] will be performed to assess the feasibility of the pharmacist service. Descriptive statistics will be employed to report demographic characteristics and health information obtained from the Resident Care Assessment Record.

For peer review only

To determine *reach*, the number and proportion of eligible residents who were willing to participate in the study will be calculated. Information that will be collected include number of residents approached by the study staff or pharmacist and provided with the study information, number of residents who meet the inclusion criteria, number of residents who provided consent, and number of participants who completed the 12-week study.

To determine *effectiveness*, changes in resident symptom score pre- and post-digital pharmacist service, as measured using the Edmonton Symptom Assessment System (ESAS), will be assessed. Changes in medication use will be assessed, including changes to sedative and anticholinergic load pre- and post-digital pharmacist service. Changes in 24-hour movement behaviour, as measured using the activity tracker, will be assessed pre- and post-digital pharmacist service. The number of adverse events including falls (both injurious and non-injurious), fractures, delirium, faecal impaction, hospitalisation or emergency department presentation due to adverse medication events, and medication incidents will be assessed. The number of adverse events experienced by residents within the 12-weeks pre-digital pharmacist service versus number of adverse events experienced during the 12-week digital pharmacist service period will be compared. Pre-post paired t-test or Wilcoxon tests will be utilised for continuous data and McNemar's tests for categorical variables.

For peer review only

To assess service *adoption*, the number and proportion of organisation staff invited, that agreed to participate, will be calculated.

To assess *implementation*, the number and types of pharmacist recommendations will be recorded and evaluated. In addition, the proportion of recommendations made by the pharmacist will be compared with the number of recommendations accepted, to determine the acceptability of the digital pharmacist service by the GPs and aged care staff. Semi-structured interviews or focus groups will be conducted with the project pharmacist, GPs, aged care staff, residents and family members to understand their views of the digital pharmacist service, including facilitators and barriers to its delivery. An analysis will be undertaken of the costs associated with implementing the ADEPT-assisted pharmacist service. The resource use associated with the implementation of the ADEPT pharmacist service, including staff (aged care and health professionals) time, equipment, and resources will be recorded by trial staff prospectively. Changes in pharmacist time spent reviewing residents will be measured using pharmacist’s record of amount of time spent reviewing medications for residents, generating recommendations and communicating with staff, GPs and residents. Resources will be valued using appropriate unit costs drawn from organisational finance data, relevant published employment awards [37], the Medicare Benefits Schedule (list of medical services that the Australian Government provides a rebate

For peer review only

to assist with costs) and the Pharmaceutical Benefits Scheme (Australian Government program that subsidises costs of prescription medications) [38, 39]. Where valuations are unable to be obtained from published data, we will value using market rates, using the average cost from three suppliers. Prices will be updated to the current year using the Consumer Price Index [40]. A budget impact analysis will be conducted, extrapolating the within-study assessment of costs to a wider population level.

To assess *maintenance*, interviews with senior management at the aged care facility will be conducted to understand intention to continue to implement the service post study funding, and whether the service aligns to the facility's mission or sustainability of business model. The capacity of the aged care organisation to implement the service (beyond the feasibility study) to identify and negate potential medicine-induced harms will be explored.

Informed consent

Prior to the conduct of any study procedures, study staff will provide residents or an authorised representative with the participant information sheet and consent form. The purpose of the study and all study procedures will be discussed with the resident or an authorised representative. Informed written consent will be obtained by the resident or

For peer review only

authorised representative. Participants will be assured that participation is completely voluntary and that they can withdraw from participation at any time.

Data management

All identifying information will be kept in secure settings in the residential aged care facility, University of South Australia (QUMPRC) or University of New South Wales, accessible only by central study staff. Where necessary, electronic tablets used for data collection (under password protection), and hard copies of questionnaires, will be stored temporarily within the residential aged care facility. The electronic tablets and forms will be stored in locked cabinets within the facility (accessible only by study staff). Information containing identifiers will be removed at the end of data collection by the project lead. The QUMPRC physical and IT environment operates to in-confidence and protected levels according to Department of Defence security standards.

Risks

The aim of the current study is to test the feasibility of using digital technologies to help pharmacists better monitor the effects of medicines that the residents are taking. There

For peer review only

are no apparent risks to participants that would be expected as a result of the pharmacist service or the unintrusive activity and sleep monitors. It is anticipated that there may be direct benefits to the participants through a reduction in adverse events.

Ethics and dissemination

Ethical approval has been obtained from the University of South Australia Human Research Ethics Committee (HREC205098). The study will be conducted in accordance with principles of the Declaration of Helsinki [41] and the National Statement of Ethical Conduct in Human Research [42]. During the study, any amendment to the study protocol or related documents will be submitted to the ethics committees for approval prior to implementation.

A Clinical Study Report in accordance with relevant ICH guidelines will be prepared, summarising all study data and analysis. Study findings may also be published or presented at scientific meetings. All publications arising from the study will comply with recognised ethical standards concerning publications and authorships. In all instances, dissemination of the study results will include de-identified or average study data and will not identify an individual participant.

For peer review only

Data availability

Individual participant data will not be made available due to data being collected from a small group of aged care residents located at one facility in South Australia, and the high risk of re-identification of individual participants.

Trial Registration number

ACTRN12623000506695

Authors' Contributions

PB, TM, DD, RM, EER and RL conceived the study and obtained study funding. MSB, PB, JS, DD, TM and RL assisted in the development of the study protocol. MSB drafted the protocol manuscript and co-ordinates the day-to-day operation of the study. PB developed the TeleClinical Care dashboard for the ADEPT study based on designs and prior development by NHL and PB. All authors critically revised and approved the final manuscript.

For peer review only

Funding statement

This project is funded by an Aged Care Research & Industry Innovation Australia (ARIIA) grant (GO00039). JS is employed by the organisation providing aged care service to the study participants. DD is supported by a Discovery Early Career Research Award (DE230101174). RL is supported by National Health and Medical Research Council Early Career Fellowship (APP1156368).

Competing interests statement

None declared.

Patient consent for publication

Not required.

For peer review only

References

1. Sluggett, J.K., et al., *Medication management policy, practice and research in Australian residential aged care: Current and future directions.* Pharmacological Research, 2017. **116**: p. 20-28.
2. Lim, R., et al., *The extent of medication-related hospital admissions in Australia: A review from 1988 to 2021.* Drug Safety, 2022. **45**(3): p. 249-257.
3. Handler, S.M., et al., *Epidemiology of medication-related adverse events in nursing homes.* The American Journal of Geriatric Pharmacotherapy, 2006. **4**(3): p. 264-272.
4. Al-Jumaili, A.A. and W.R. Doucette, *A systems approach to identify factors influencing adverse drug events in nursing homes.* Journal of the American Geriatrics Society, 2018. **66**(7): p. 1420-1427.
5. Tenhunen, M.L., B. Smithers, and B. Tucker, *Identifying medication-related adverse drug events in nursing facilities in east Texas.* The Consultant Pharmacist, 2016. **31**(8): p. 436-439.
6. Kalisch Ellett, L.M., et al., *Prevalence and Preventability of Adverse Medicine Events in a Sample of Australian Aged-Care Residents: A Secondary Analysis of Data from the ReMInDAR Trial.* Drug Safety, 2023. **46**(5): p. 493-500.
7. Sluggett, J.K., et al., *Provision of a comprehensive medicines review is associated with lower mortality risk for residents of aged care facilities: a retrospective cohort study.* Age and Ageing, 2022. **51**(7).
8. Australian Government Department of Health. *Aged care on-site pharmacist.* 2023; Available from: https://www.health.gov.au/our-work/aged-care-on-site-pharmacist.
9. Roughead, E.E., et al., *Effect of an ongoing pharmacist service to reduce medicine-induced deterioration and adverse reactions in aged-care facilities (nursing homes): a

For peer review only

multicentre, randomised controlled trial (the ReMInDAR trial). Age and Ageing, 2022. **51**(4).

10. Lim, R., et al., *Reducing medicine-induced deterioration and adverse reactions (ReMInDAR) trial: study protocol for a randomised controlled trial in residential aged-care facilities assessing frailty as the primary outcome.* 2020. **10**(4): p. e032851.

11. Lim, R., et al., *Pharmacists as patient advocates: A series of case studies illustrating the impacts of a regular pharmacist service in residential aged care (nursing homes).* Exploratory Research in Clinical and Social Pharmacy, 2023. **10**: p. 100268.

12. Pratt, N.L., et al., *Association between use of multiple psychoactive medicines and hospitalization for falls: retrospective analysis of a large healthcare claim database.* Drug Safety, 2014. **37**(7): p. 529-35.

13. Lim, R., et al., *Using wrist-worn accelerometers to identify the impact of medicines with anticholinergic or sedative properties on sedentary time: A 12-month prospective analysis.* Maturitas, 2023. **172**: p. 9-14.

14. Theou, O., et al., *Association between sedentary time and mortality across levels of frailty.* Canadian Medical Associtionn Journal, 2017. **189**(33): p. E1056-e1064.

15. Indraratna, P., et al., *Trials and tribulations: mHealth clinical trials in the COVID-19 pandemic.* Yearbook of Medical Informatics, 2021. **30**(1): p. 272-279.

16. Indraratna, P., et al., *A smartphone-based model of care to support patients with cardiac disease transitioning from hospital to the community (TeleClinical Care): Pilot randomized controlled trial.* Journal of Medical Internet Research, 2022. **10**(2): p. e32554.

For peer review only

17. Hui, D. and E. Bruera, *The Edmonton Symptom Assessment System 25 years later: Past, present, and future developments.* Journal of Pain Symptom Management, 2017. **53**(3): p. 630-643.

18. Fibion. *Fibion SENS*. 2020; Available from: https://sens.fibion.com/.

19. Fibion. *Fibion Emfit*. 2022; Available from: https://emfit.fibion.com/.

20. Roughead, E., et al., *Reducing harms from medicines in aged-care: findings from the ReMInDAR trial.* 2021, The University of South Australia.

21. Leecare, *P6Med*. 2023.

22. Medi-Map Group Pty Ltd, *Medi-Map*. 2023.

23. Nasreddine, Z.S., et al., *The Montreal Cognitive Assessment, MoCA: A brief screening tool for mild cognitive impairment.* Journal of the American Geriatrics Society, 2005. **53**(4): p. 695-699.

24. Ghaffari, A., et al., *Criterion calidity of linear accelerations measured with low-sampling-frequency accelerometers during overground walking in elderly patients with knee osteoarthritis.* Sensors, 2022. **22**(14): p. 5289.

25. Pedersen, B.S., et al., *Validation of two activity monitors in slow and fast walking hospitalized patients.* Rehabilitation Research and Practice, 2022. **2022**: p. 9230081.

26. Zink, M.D., et al., *Unobtrusive nocturnal heartbeat monitoring by a ballistocardiographic sensor in patients with sleep disordered breathing.* Scientific Reports, 2017. **7**(1): p. 13175.

27. Buimer, H.P., et al., *Sleep–wake monitoring of people with intellectual disability: Examining the agreement of EMFIT QS and actigraphy.* Journal of Applied Research in Intellectual Disabilities, 2023.

For peer review only

28. Veterans' MATES. *Cumulative risk calculator*. 2022; Available from: https://www.veteransmates.net.au/cumulative-risk-calculator.

29. Mankowski, R.T., et al., *Device-measured physical activity as a predictor of disability in mobility-limited older adults.* Journal of the American Geriatrics Society, 2017. **65**(10): p. 2251-2256.

30. Vincent, B.M., et al., *Sleeping time is associated with functional limitations in a national sample of older Americans.* Aging Clinical and Experimental Research, 2021. **33**(1): p. 175-182.

31. Goldman, S.E., et al., *Poor sleep is associated with poorer physical performance and greater functional limitations in older women.* Sleep, 2007. **30**(10): p. 1317-24.

32. Guzman, H., et al., *Clinically significant blood pressure reduction among participants of a lifestyle intervention improvement program strongly associated with reduced systemic inflammation in a high risk working population.* Circulation, 2014. **129**(suppl_1): p. AP391-AP391.

33. Williamson, D.A., G.A. Bray, and D.H. Ryan, *Is 5% weight loss a satisfactory criterion to define clinically significant weight loss?* Obesity 2015. **23**(12): p. 2319-20.

34. Eldridge, S.M., et al., *CONSORT 2010 statement: extension to randomised pilot and feasibility trials.* BMJ, 2016. **355**: p. i5239.

35. Sim, J. and M. Lewis, *The size of a pilot study for a clinical trial should be calculated in relation to considerations of precision and efficiency.* Journal of Clinical Epidemiology, 2012. **65**(3): p. 301-8.

36. Glasgow, R.E., et al., *RE-AIM planning and evaluation framework: Adapting to new science and practice with a 20-year review.* Frontiers in Public Health, 2019. **7**: p. 64.

For peer review only

37. Australian Government Fair Work Ombudsman. *Fair Work Ombudsman*. Available from: https://www.fairwork.gov.au/.

38. Australian Government Department of Health and Aged Care. *MBS Online contains the Medicare Benefits Schedule (MBS), a listing of the Medicare services subsidised by the Australian Government*. 2023; Available from: http://www.mbsonline.gov.au/internet/mbsonline/publishing.nsf/Content/Home.

39. Australian Government Department of health and Aged Care. *Pharmaceutical Benefits Scheme (PBS)*. 2023; Available from: https://www.pbs.gov.au/pbs/home.

40. Australian Bureau of Statistics. *Price indexes and inflation*. 2023.

41. World Medical Association, *World medical association declaration of Helsinki: Ethical principles for medical research involving human subjects.* JAMA, 2013. **310**(20): p. 2191-2194.

42. National Health and Medical Research Council. *National Statement of Ethical Conduct in Human Research.* 2007; Available from: https://www.nhmrc.gov.au/about-us/publications/national-statement-ethical-conduct-human-research-2007-updated-2018.

Figure 1A shows example vital signs (blood pressure, body weight, and oxygen saturation) with onset of a new medication (Alprazolam 0.25 mg tds) overlaid in the TCC-ADEPT platform. Figure 1B shows example cumulative risks and type of adverse drug reactions displayed in the TCC-ADEPT platform.

Figure 2. Proposed service flow

For peer review only

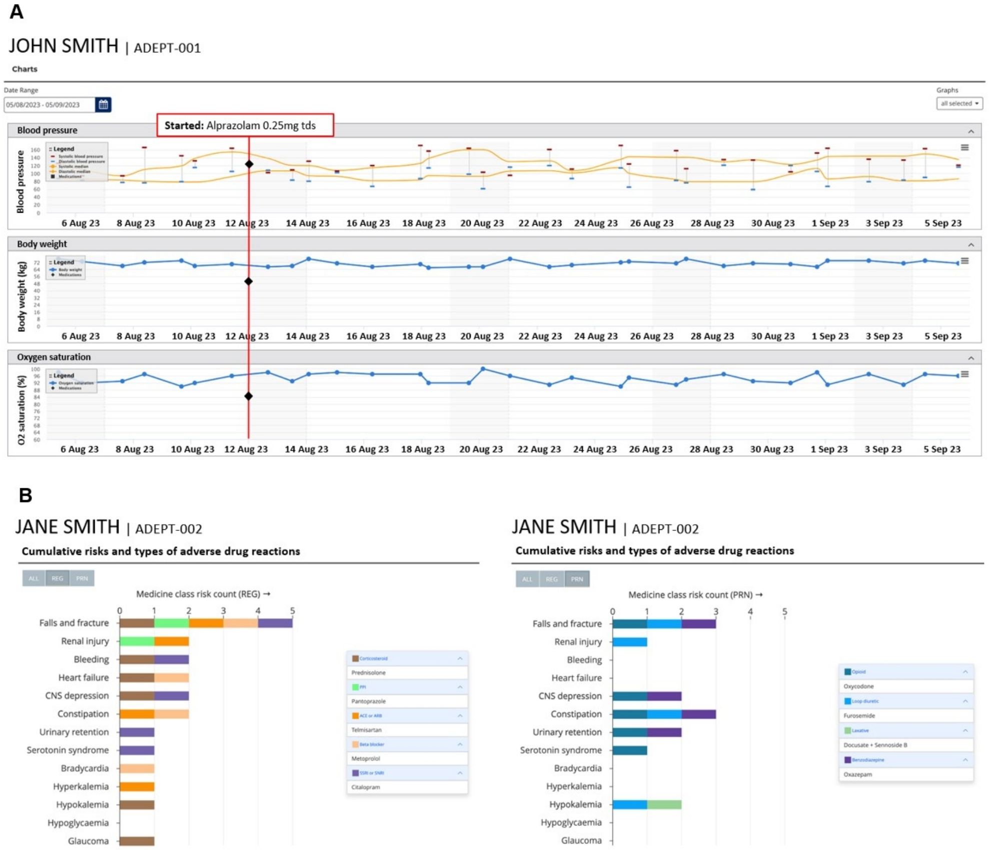

Figure 1A shows example vital signs (blood pressure, body weight, and oxygen saturation) with onset of a new medication (Alprazolam 0.25 mg tds) overlaid in the TCC-ADEPT platform. Figure 1B shows example cumulative risks and type of adverse drug reactions displayed in the TCC-ADEPT platform.

223x194mm (144 x 144 DPI)

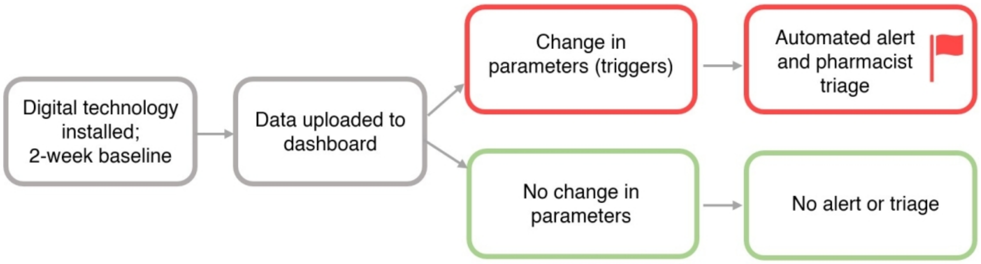

Figure 2. Proposed service flow

277x81mm (144 x 144 DPI)

University of South Australia

Consent Form

This project has been approved by the University of South Australia's Human Research Ethics Committee. If you have any ethical concerns about the project, or questions about your rights as a participant, please contact the Executive Officer of this Committee, Tel: +61 8 8302 6330; Email: humanethics@unisa.edu.au

SECTION 1: CONTACT AND PROJECT DETAILS	
Researcher's Full Name	[Chief Investigator]
Contact Details	[Phone number]
Project Number	ADEPT-2022.1.0 (Ethics ID 205098)
Project Title	A Digitally Enabled, Pharmacist service to detecT medicine harms in residential aged care: the ADEPT project

SECTION 2: PARTICIPANT CERTIFICATION

In signing this form, I confirm that:

- I have read the Participant Information Sheet, and the nature and the purpose of the research project has been explained to me. I understand and agree to take part.
- I understand the nature of my involvement in the project.
- I understand that I may not directly benefit from taking part in the project.
- I understand that I can withdraw from the project at any stage and that this will not affect my status now or in the future.
- I understand that data will be stored in the secure settings in the Quality Use of Medicines and Pharmacy Research Centre, University of South Australia. I understand that all paper and electronic records collected as part of the project will be kept for 7 years after final publication of project results.
- I understand that while information gained during the project may be published, I will not be identified and my personal results will remain confidential, unless required by law.
- I understand that data collected from this project may be used for another purpose by the researcher for which ethics approval will be sought.

Participant's Signature	*Printed Name*	*Date*
Legal guardian's Signature (if signing of behalf of participant)	*Printed Name*	*Date*

SECTION 3: RESEARCHER CERTIFICATION

I have explained the study to the participant and/or legal guardian and consider that he/she understand what is involved.

Researcher Signature	*Printed Name*	*Date*

For peer review only - http://bmjopen.bmj.com/site/about/guidelines.xhtml

SPIRIT
STANDARD PROTOCOL ITEMS: RECOMMENDATIONS FOR INTERVENTIONAL TRIALS

SPIRIT 2013 Checklist: Recommended items to address in a clinical trial protocol and related documents*

Section/item	Item No	Description	Addressed on page number
**Administrative information**			
Title	1	Descriptive title identifying the study design, population, interventions, and, if applicable, trial acronym	______1______
Trial registration	2a	Trial identifier and registry name. If not yet registered, name of intended registry	______3______
	2b	All items from the World Health Organization Trial Registration Data Set	_____N/A_____
Protocol version	3	Date and version identifier	_____N/A_____
Funding	4	Sources and types of financial, material, and other support	_____21______
Roles and responsibilities	5a	Names, affiliations, and roles of protocol contributors	____1 & 20____
	5b	Name and contact information for the trial sponsor	_____21______
	5c	Role of study sponsor and funders, if any, in study design; collection, management, analysis, and interpretation of data; writing of the report; and the decision to submit the report for publication, including whether they will have ultimate authority over any of these activities	_____N/A_____
	5d	Composition, roles, and responsibilities of the coordinating centre, steering committee, endpoint adjudication committee, data management team, and other individuals or groups overseeing the trial, if applicable (see Item 21a for data monitoring committee)	_____N/A_____

For peer review only - http://bmjopen.bmj.com/site/about/guidelines.xhtml

Introduction

Background and rationale	6a	Description of research question and justification for undertaking the trial, including summary of relevant studies (published and unpublished) examining benefits and harms for each intervention	____4 & 5_____
	6b	Explanation for choice of comparators	______19_____
Objectives	7	Specific objectives or hypotheses	____5 & 6_____
Trial design	8	Description of trial design including type of trial (eg, parallel group, crossover, factorial, single group), allocation ratio, and framework (eg, superiority, equivalence, noninferiority, exploratory)	______6______

Methods: Participants, interventions, and outcomes

Study setting	9	Description of study settings (eg, community clinic, academic hospital) and list of countries where data will be collected. Reference to where list of study sites can be obtained	______7______
Eligibility criteria	10	Inclusion and exclusion criteria for participants. If applicable, eligibility criteria for study centres and individuals who will perform the interventions (eg, surgeons, psychotherapists)	______7______
Interventions	11a	Interventions for each group with sufficient detail to allow replication, including how and when they will be administered	___11 – 14____
	11b	Criteria for discontinuing or modifying allocated interventions for a given trial participant (eg, drug dose change in response to harms, participant request, or improving/worsening disease)	_____17______
	11c	Strategies to improve adherence to intervention protocols, and any procedures for monitoring adherence (eg, drug tablet return, laboratory tests)	____18 & 19____
	11d	Relevant concomitant care and interventions that are permitted or prohibited during the trial	______7______
Outcomes	12	Primary, secondary, and other outcomes, including the specific measurement variable (eg, systolic blood pressure), analysis metric (eg, change from baseline, final value, time to event), method of aggregation (eg, median, proportion), and time point for each outcome. Explanation of the clinical relevance of chosen efficacy and harm outcomes is strongly recommended	___15 – 17____
Participant timeline	13	Time schedule of enrolment, interventions (including any run-ins and washouts), assessments, and visits for participants. A schematic diagram is highly recommended (see Figure)	____7 – 10____

For peer review only - http://bmjopen.bmj.com/site/about/guidelines.xhtml

Sample size	14	Estimated number of participants needed to achieve study objectives and how it was determined, including clinical and statistical assumptions supporting any sample size calculations	_____15______
Recruitment	15	Strategies for achieving adequate participant enrolment to reach target sample size	____7 & 15_____

Methods: Assignment of interventions (for controlled trials)

Allocation:

Sequence generation	16a	Method of generating the allocation sequence (eg, computer-generated random numbers), and list of any factors for stratification. To reduce predictability of a random sequence, details of any planned restriction (eg, blocking) should be provided in a separate document that is unavailable to those who enrol participants or assign interventions	_____N/A_____
Allocation concealment mechanism	16b	Mechanism of implementing the allocation sequence (eg, central telephone; sequentially numbered, opaque, sealed envelopes), describing any steps to conceal the sequence until interventions are assigned	_____N/A_____
Implementation	16c	Who will generate the allocation sequence, who will enrol participants, and who will assign participants to interventions	_____N/A_____
Blinding (masking)	17a	Who will be blinded after assignment to interventions (eg, trial participants, care providers, outcome assessors, data analysts), and how	_____N/A_____
	17b	If blinded, circumstances under which unblinding is permissible, and procedure for revealing a participant's allocated intervention during the trial	_____N/A_____

Methods: Data collection, management, and analysis

Data collection methods	18a	Plans for assessment and collection of outcome, baseline, and other trial data, including any related processes to promote data quality (eg, duplicate measurements, training of assessors) and a description of study instruments (eg, questionnaires, laboratory tests) along with their reliability and validity, if known. Reference to where data collection forms can be found, if not in the protocol	___7 – 11_____
	18b	Plans to promote participant retention and complete follow-up, including list of any outcome data to be collected for participants who discontinue or deviate from intervention protocols	_____N/A_____

Data management	19	Plans for data entry, coding, security, and storage, including any related processes to promote data quality (eg, double data entry; range checks for data values). Reference to where details of data management procedures can be found, if not in the protocol	___11 & 17____
Statistical methods	20a	Statistical methods for analysing primary and secondary outcomes. Reference to where other details of the statistical analysis plan can be found, if not in the protocol	___15 – 17____
	20b	Methods for any additional analyses (eg, subgroup and adjusted analyses)	_____N/A_____
	20c	Definition of analysis population relating to protocol non-adherence (eg, as randomised analysis), and any statistical methods to handle missing data (eg, multiple imputation)	_____N/A_____
**Methods: Monitoring**			
Data monitoring	21a	Composition of data monitoring committee (DMC); summary of its role and reporting structure; statement of whether it is independent from the sponsor and competing interests; and reference to where further details about its charter can be found, if not in the protocol. Alternatively, an explanation of why a DMC is not needed	_____N/A_____
	21b	Description of any interim analyses and stopping guidelines, including who will have access to these interim results and make the final decision to terminate the trial	_____N/A_____
Harms	22	Plans for collecting, assessing, reporting, and managing solicited and spontaneously reported adverse events and other unintended effects of trial interventions or trial conduct	_____19______
Auditing	23	Frequency and procedures for auditing trial conduct, if any, and whether the process will be independent from investigators and the sponsor	_____N/A_____
**Ethics and dissemination**			
Research ethics approval	24	Plans for seeking research ethics committee/institutional review board (REC/IRB) approval	_____19______
Protocol amendments	25	Plans for communicating important protocol modifications (eg, changes to eligibility criteria, outcomes, analyses) to relevant parties (eg, investigators, REC/IRBs, trial participants, trial registries, journals, regulators)	___19 – 20____

Consent or assent	26a	Who will obtain informed consent or assent from potential trial participants or authorised surrogates, and how (see Item 32)	_____17______
	26b	Additional consent provisions for collection and use of participant data and biological specimens in ancillary studies, if applicable	_____N/A_____
Confidentiality	27	How personal information about potential and enrolled participants will be collected, shared, and maintained in order to protect confidentiality before, during, and after the trial	____17 & 20____
Declaration of interests	28	Financial and other competing interests for principal investigators for the overall trial and each study site	_____21______
Access to data	29	Statement of who will have access to the final trial dataset, and disclosure of contractual agreements that limit such access for investigators	_____17______
Ancillary and post-trial care	30	Provisions, if any, for ancillary and post-trial care, and for compensation to those who suffer harm from trial participation	_____N/A_____
Dissemination policy	31a	Plans for investigators and sponsor to communicate trial results to participants, healthcare professionals, the public, and other relevant groups (eg, via publication, reporting in results databases, or other data sharing arrangements), including any publication restrictions	_____20______
	31b	Authorship eligibility guidelines and any intended use of professional writers	_____20______
	31c	Plans, if any, for granting public access to the full protocol, participant-level dataset, and statistical code	_____20______
**Appendices**			
Informed consent materials	32	Model consent form and other related documentation given to participants and authorised surrogates	supplemental material
Biological specimens	33	Plans for collection, laboratory evaluation, and storage of biological specimens for genetic or molecular analysis in the current trial and for future use in ancillary studies, if applicable	_____N/A_____

*It is strongly recommended that this checklist be read in conjunction with the SPIRIT 2013 Explanation & Elaboration for important clarification on the items. Amendments to the protocol should be tracked and dated. The SPIRIT checklist is copyrighted by the SPIRIT Group under the Creative Commons "Attribution-NonCommercial-NoDerivs 3.0 Unported" license.

For peer review only - http://bmjopen.bmj.com/site/about/guidelines.xhtml

BMJ Open

A Digitally Enabled, Pharmacist service to detecT medicine harms in residential aged care (nursing home) (ADEPT): protocol for a feasibility study.

Journal:	*BMJ Open*
Manuscript ID	bmjopen-2023-080148.R2
Article Type:	Protocol
Date Submitted by the Author:	29-Jan-2024
Complete List of Authors:	Boord, Monique; University of South Australia, Quality Use of Medicines and Pharmacy Research Centre<br>Brown, Peter; University of New South Wales, Tyree Foundation Institute of Health Engineering (IHealthE) and Graduate School of Biomedical Engineering<br>Soriano, Julian; Tanunda Lutheran Home Inc; SA Pharmacy<br>Meola, Tahlia; University of South Australia, UniSA Clinical and Health Sciences<br>Dumuid, Dorothea ; University of South Australia, Alliance for Research in Exercise, Nutrition and Activity<br>Milte, Rachel; Flinders University, Rehabilitation, Aged and Extended Care<br>Roughead, Elizabeth; University of South Australia, Quality Use of Medicines and Pharmacy Research Centre, UniSA Clinical and Health Sciences<br>Lovell, Nigel; University of New South Wales, Tyree Foundation Institute of Health Engineering (IHealthE) and Graduate School of Biomedical Engineering<br>Stone, Helen; Pharmaceutical Society of Australia<br>Whitehouse, Joseph; HealthyCare Services<br>Janetzki, Jack; University of South Australia, Quality Use of Medicines and Pharmacy Research Centre; University of South Australia, UniSA Clinical and Health Sciences<br>Gebreyohannes, Eyob Alemayehu; University of South Australia, Quality Use of Medicines and Pharmacy Research Centre, UniSA Clinical and Health Sciences; The University of Western Australia, School of Allied Health<br>Lim, Renly; University of South Australia, Quality Use of Medicines and Pharmacy Research Centre, UniSA Clinical and Health Sciences
**Primary Subject Heading**:	Geriatric medicine
Secondary Subject Heading:	Health services research
Keywords:	Adverse events < THERAPEUTICS, Aged, Health Services for the Aged, GERIATRIC MEDICINE, Health policy < HEALTH SERVICES ADMINISTRATION & MANAGEMENT, Patient-Centered Care

SCHOLARONE™
Manuscripts

A Digitally Enabled, Pharmacist service to detecT medicine harms in residential aged care (nursing home) (ADEPT): protocol for a feasibility study.

Authors

Monique S Boord[1], Peter Brown[2], Julian Soriano[3,4], Tahlia Meola[5], Dorothea Dumid[6], Rachel Milte[7], Elizabeth E Roughead[1], Nigel H. Lovell[2], Helen Stone[8], Joseph Whitehouse[9], Jack L Janetzki[1,5], Eyob Alemayehu Gebreyohannes[1,10], Renly Lim[1]

Institutions

[1] Quality Use of Medicines and Pharmacy Research Centre, UniSA Clinical and Health Sciences, University of South Australia, Adelaide, Australia

[2] Tyree Foundation Institute of Health Engineering (IHealthE) and Graduate School of Biomedical Engineering, UNSW, Sydney, Australia

[3] Tanunda Lutheran Home, Tanunda, Australia

[4] SA Pharmacy, Adelaide, Australia

[5] UniSA Clinical and Health Sciences, University of South Australia, Adelaide, Australia

[6] Alliance for Research in Exercise, Nutrition and Activity, UniSA Clinical and Health Sciences, University of South Australia, Adelaide, Australia

For peer review only

[7] Caring Futures Institute, College of Nursing and Health Sciences, Flinders University, Adelaide, Australia

[8] Pharmaceutical Society of Australia, Deakin, Australia

[9] HealthyCare Services, Welland, Australia

[10] School of Allied Health, The University of Western Australia, Perth, Australia

Correspondence to:

Dr Monique Boord

Quality Use of Medicines and Pharmacy Research Centre,

UniSA Clinical and Health Sciences,

University of South Australia, Adelaide, Australia

Monique.boord@unisa.edu.au

Abstract

Introduction

This feasibility study aims to develop and test a new model of practice in Australia: using digital technologies to enable pharmacists to monitor early signs and symptoms of medicine-induced harms in residential aged care.

For peer review only

Methods and analysis

Thirty residents will be recruited from an aged care facility in South Australia. The study will be conducted in two phases. In phase 1, the study team will work with aged care software providers and developers of digital technologies (a wearable activity tracker and a sleep tracking sensor) to gather physical activity and sleep data, as well as medication and clinical data from the electronic medication management system and aged care clinical software. Data will be centralised into a cloud-based monitoring platform (TeleClinical Care). The TeleClinical Care (TCC) will be used to create dashboards that will include longitudinal visualisations of changes in residents' health, function, and medicine use over time. In Phase 2, the on-site pharmacist will use the centralised TCC platform to monitor each resident's medicine, clinical, physical activity and sleep data to identify signs of medicine-induced harms over a 12-week period.

A mixed methods process evaluation applying the RE-AIM evaluation framework will be used to assess the feasibility of the service. Outcome measures include service reach, changes in resident symptom scores (measured using the Edmonton Symptom Assessment System), number of medication adverse events detected, changes in physical activity and

For peer review only

sleep, number of pharmacist recommendations provided, cost analysis, and proportion of all pharmacists' recommendations implemented at 4-, 8-, and 12-weeks post-baseline period.

Ethics and dissemination

Ethical approval has been obtained from the University of South Australia's Human Research Ethics Committee (205098). Findings will be disseminated through published manuscripts, conference presentations, and reporting to the study funder.

Trial registration number

ACTRN12623000506695

Strengths and limitations of this study

- Combined various sources of health data into a single centralised platform for the residential aged care pharmacist to actively monitor medicine-induced harms.
- Uses objective assessments of changes in physical activity and sleep to identify "mild" medicine harms.
- Due to recruiting from a single residential aged care facility in South Australia, findings may not generalise across all residents living in aged care.

For peer review only

- Lack of compliance with use and wearing of digital technologies by residents is a potential limitation.

For peer review only

Introduction

Approximately eight percent of adults in Australia aged 65 and older reside in aged-care facilities (nursing home) [1], with up to 63% receiving nine or more regular medications [1]. Medicine-related problems contribute to an estimated 250,000 hospital admissions in Australia every year, with an estimated annual cost of $1.4 billion [2]. Medicine-related harms are of significant concern in residential aged care, with up to 27 adverse medicine events occurring per 100 residents per month [3-5]. In Australia, about one in five persons living in residential aged care experience an adverse medicine event every month; of which 83% were potentially preventable [6]. Despite the significant medicine-related harms, most Australian residential aged care facilities have limited capacity to provide clinical pharmacy services; existing services are limited to government funded residential medication management review (comprehensive medication reviews performed by accredited pharmacists in residential aged care) and quality use of medicine services [7].

The Australian Government, recognising the significance of medicine-related problems in aged care, committed $350 million to improve medication safety in residential aged care through funding for pharmacists [8]. The government investment presents new opportunities for pharmacists to review residents for signs and symptoms of medicine harms more frequently. The present study builds on our recent randomised controlled trial which

For peer review only

investigated the effectiveness of ongoing pharmacist assessments compared with usual care in reducing medicine induced deterioration, frailty, and adverse events across 39 residential aged care facilities in Australia [9, 10]. Our study showed that one in five residents experienced a preventable adverse event (e.g., falls, fractures) every month due to medication use [9]. The pharmacists identified and resolved medicine-related harms and served as an advocate for residents. Further, significant differences in change in cognition from baseline were observed between intervention and control groups [9]. The service ensured pharmacist engagement with residents, rather than just reviewing medicines, which enabled the advocacy [11].

Frequent monitoring of medicine-induced harms is important because medicines can cause mild harm (e.g., sedation, poor balance) that often remains undetected, but can lead to serious adverse events in older people if left unmanaged. For example, commonly prescribed psychotropic medicines can cause sedation and poor cognition that may lead to injurious falls and fractures [12]. An increase of sedative load from two to four over a 12-month period has been associated with an average increase in daily sedentary behaviour of approximately 24 minutes [13]; an increase of 60 minutes per day of sedentary time is associated with a 19% increase in risk of death [14]. Detecting mild, cumulative effects of

For peer review only

110 medicine-induced harms, including changes in physical activity, is likely to be important,
111 however, this can be difficult and labour intensive to measure and monitor.

112 Harnessing digital technologies to enable active monitoring of medicine-related harms is
113 one potential strategy to enabling proactive monitoring. For pharmacists to effectively
114 monitor harms, they will not only need to review medicines, but also recognise and monitor
115 early signs of medicine-induced harms such as changes in sleep, activity, or cognition [9,
116 13]. Digital technologies can measure changes in sleep, activity, or cognition objectively,
117 which may enhance effective communication about medicines-related harm across aged
118 care residents' care teams, including to general practitioners (GPs), nurses and care staff,
119 as well as support the recommendations from pharmacists and their acceptance by the team
120 and residents themselves. The TeleClinical-Care (TCC) platform has been used successfully
121 during the COVID-19 pandemic to safely manage more than 8,000 COVID-positive patients
122 as they self-isolated at home [15] and is being used in trials to manage cardiovascular
123 disease and stroke [16].

124 The ADEPT project aims to develop and implement a digitally enabled, evidence-based,
125 pharmacist service built upon TCC (TCC-ADEPT) to actively detect medicine harms in
126 residential aged care. Specifically, we will:

For peer review only

1. Determine the number and proportion of eligible residents who were willing to participate in the study;
2. Determine changes in resident outcomes, including changes in symptoms as measured using the Edmonton Symptom Assessment System (ESAS) [17], physical activity and sleep (24-hour movement behaviour), and adverse events including falls (both injurious and non-injurious), fractures, delirium, faecal impaction, hospitalisation or emergency department presentation due to adverse medication events, and medication incidents, pre- and post-digital pharmacist service;
3. Determine the number and types of pharmacist recommendations that resulted following use of the TCC-ADEPT platform, and the number of pharmacist recommendations that were accepted and implemented by GPs and aged care staff;
4. Explore the acceptability of the digital pharmacist service by residents and family members, aged care staff and GPs;
5. Assess the costs associated with implementing the TCC-ADEPT platform and potential cost impacts to the health and aged care system in wider-spread roll out of the platform;

For peer review only

6. Explore the residential aged care facility's intention to continue to implement the pharmacist service post study funding and whether the service aligns to the facility's mission or sustainability of business model.

We hypothesise that the new pharmacist service is feasible and acceptable to aged care staff, residents and their GPs, and can enhance detection of early signs and symptoms of medicine harms, triggering review and action when required.

Methods and analysis

Study design

The ADEPT project will be conducted in two phases over a 12-month period. Phase one will include gathering data from digital technologies, medication and health records into the TCC platform. Digital technologies will include an activity tracker [18] and a sleep sensor [19]. A pre-post12-week implementation study will constitute phase two of the project. The on-site pharmacist will monitor all participants using the centralised TCC-ADEPT platform, using data to inform resident reviews and to initiate conversations with residents, GPs and aged care staff on medicine, health and quality of life changes for residents. Recruitment and study implementation will occur from August 2023 until February 2024.

For peer review only

Patient and public involvement

Aged care residents, representatives and health professionals were directly involved in the development or design of this study. Findings from interviews with residents, carers and aged care staff from the ReMInDAR trial were used to inform the development and design of this study [20].

Cohort selection

Residents will be considered eligible to participate if they are a permanent resident living in the designated residential aged care facility. Exclusion criteria include receiving palliative or respite care.

Screening and recruitment

Residents will be recruited from an aged care facility in South Australia. Promotional recruitment material (flyers) will be distributed onsite in common areas of the facility one month prior to study commencement. Participant information sheets will be provided to residents deemed potentially eligible by facility staff, and will be given to all residents willing to participate, informing them of the study details and pharmacist service.

Study staff will perform initial screening of residents' care record to identify potentially eligible residents. Screening logs will be kept monitoring residents' recruitment and to enable

For peer review only

a description of the study population from which eligible residents have been enrolled. If the resident fulfills inclusion criteria based on the initial screening process, the on-site pharmacist will invite the resident to participate in the study and written informed consent will be obtained from the resident or their authorised representative (model consent form shown in Supplementary Material).

Baseline data collection

Once written consent is obtained, study staff will collect baseline data from the Resident Care Assessment Record held electronically in Leecare [21] and Medi-Map [22] software, which contains demographic details, comorbidities, medicine use, falls history, and care provided within the aged care facility. Study staff will administer the Edmonton Symptom Assessment Scale (ESAS) [17] and the Montreal Cognitive Assessment (MoCA) [23] at baseline. An observational logbook or diary will be kept to record information not collected in the existing data fields. Table 1 indicates data collected at each data collection time points.

For peer review only

Table 1. Data collection schedule

Variable	Description	Data source	Data collection time points			
			Baseline	4-weeks	8-weeks	12-weeks
**Demographic**	Age, gender	Leecare	✓			
**Medical conditions**	Diagnosis of medical condition	Leecare	✓	✓	✓	✓
**Medications***	Name, strength, dose, frequency of medicine administration	Medi-Map	✓	✓	✓	✓
**Nutritional status**	Swallowing status	Leecare	✓			✓
**Functional ability**	Mobilisation status	Leecare	✓			✓
**Cognitive function**	Montreal Cognitive Assessment	Leecare	✓			✓
**Falls history**	Number of falls and related health outcomes	Leecare	✓			✓
**Vital signs**	Height, weight, blood pressure (systolic and diastolic), respirations, pulse, BGL, SpO2	Leecare	✓	✓	✓	✓
**Pain**	PainChek® score severity across 6 domains	Leecare	✓	✓	✓	✓
**Residential Medication Management Review (RMMR)**	Dates of RMMR provision will be recorded	On-site aged care pharmacist	✓	✓	✓	✓
**Physical activity**	Time spent in light, moderate, vigorous-intensity activity	Fibion SENS device	✓	✓	✓	✓
**Sleep**	Movements during sleep, durations of light, deep and REM sleep	Fibion Emfit device	✓	✓	✓	✓
**Edmonton Symptom Assessment Scale (ESAS)**	Severity across 10 symptoms	Participant or third-party informant review	✓	✓	✓	✓
**Adverse events**	E.g., falls, fracture, delirium, faecal impaction, incontinence	Leecare, Pharmacists,	✓	✓	✓	✓

For peer review only

		aged care staff, residents				
**Incidents**	Medication incidents will be recorded including date/time and severity	Leecare, Pharmacists, aged care staff, residents	✓	✓	✓	✓
**Ambulance call-outs**	Date and reasons will be recorded	Leecare, Pharmacists, aged care staff, residents	✓	✓	✓	✓
**Hospital visits**	Date and reason of emergency department visits or hospital admission will be recorded	Leecare, Pharmacists, aged care staff, residents	✓	✓	✓	✓
**All-cause mortality**	Any deaths in the 12-week study period will be recorded	Aged care staff		✓	✓	✓

Note* data will be collected and exported daily into the TCC-ADEPT platform over the 12-week study period. BGL= Blood glucose level; SpO2= oxygen saturation; REM= rapid eye movement.

For peer review only

Measures

Edmonton Symptom Assessment Scale (ESAS)

The ESAS is a 10-item numeric rating scale assessing self-reported pain, fatigue, nausea, depression, anxiety, drowsiness, shortness of breath, appetite, wellbeing, and sleep [17]. Participants or their carer will rate the severity of each symptom on a scale of 0-10 by circling a number on each scale which best describes their symptoms over the past 24 hours [17]. Higher scores indicate higher symptom burden.

Montreal Cognitive Assessment (MoCA)

The MoCA is a 1 page, 30-point screening instrument that assesses cognitive domains of memory, language, executive functions, visuospatial skills, calculation, abstraction, attention, concentration, and orientation [23]. A cut off score of 26 has been shown to differentiate mild cognitive impairment or dementia from normal cognition [23]. When scoring, a one-point adjustment is given to those with 12 or less years of formal education [23].

Fibion SENS

The activity tracker is the Fibion SENS device (SENS Motion®, Copenhagen, Denmark) [18] which is a waterproof 3D triaxial accelerometer (4.7 cm x 2.2 cm x 0.45 cm) previously validated for use in older adult samples [24, 25]. The SENS device samples data at a rate of 12.5 Hz and an algorithm categorises data into predefined activities: lying/sitting rest; lying/sitting movement; standing; walking; sporadic walking; cycling & similar of light, moderate, and vigorous intensity. Residents will be asked to continually wear the tracker if possible, or for a minimum of 16 hours per day to ensure sufficient data collection. The manufacturer recommends attaching the device to the lateral distal thigh with a medical adhesive patch approximately 10 cm above the lateral epicondyle.

For peer review only

Fibion Emfit

The sleep sensor is the Fibion Emfit sensor (Emfit Corp. Kuopio, Finland) [19]. The device is a thin, flexible under-mattress device (32 cm x 62 cm x 0.4 cm) comprised of charged polymer layers and air voids, the position of which changes with mechanical pressure to measure body movement [26, 27]. The ballistic forces generated collect activity and physiological data during sleep including sleep time, sleep classes, heart rate, breathing rate, movement and bed occupancy and exits [27].

Phase one

Dashboard integration

A protocol will be developed for extracting data from digital technologies (wearable activity trackers, and sleep tracking sensors) and resident clinical information and medication records into the TCC-ADEPT platform. The TCC-ADEPT platform will include a dashboard with longitudinal visualisations of changes in residents' health and wellbeing over time (i.e., physical activity, sleep quality, blood pressure etc) using data loaded from the digital technologies and clinical data (see Figure 1). Medicine use (including sedative load and

For peer review only

234 cumulative medicines risk calculator) and pathology records (e.g., renal function) will also be

235 uploaded to the dashboard. The platform infrastructure will be hosted on the Microsoft Azure

236 cloud service with built-in regulatory compliance for the Health Insurance Portability and

237 Accountability Act (HIPAA), and the Australian Government Information Security Manual

238 (ISM). The resulting dashboard will be operated by the on-site pharmacist in phase two

239 (feasibility study).

240

241 FIGURE 1

For peer review only

Identification of triggers for review

To determine whether a resident has experienced medicine-induced harm, the on-site pharmacists will review data from the digital technologies, residents' clinical and medicine data presented in the TCC-ADEPT dashboard. Baseline data collected within the first two weeks of the study will be averaged and serve as the reference point, and the dashboard will present longitudinal data and trends for each resident. To determine whether changes in health and function are medicine-induced, visualisations will be presented relative to each time point where medicines are added, ceased, or modified.

In-built data analytics running an automated rules-based algorithm will be used for detection of changes in physiological or other health parameters. If and when residents' health declines beyond a certain threshold compared to baseline levels, the system will automatically flag residents that will alert the pharmacist (see Figure 2).

FIGURE 2

The trigger threshold will be determined based on minimum clinically significant difference where known in the literature (see Table 2). Where minimum clinically significant

For peer review only

differences are not known, a change of 10% will be considered a trigger, or in consultation with our clinicians.

Table 2. List of triggers

	Trigger (change from participants baseline value)
**Medicine data**	Sedative load increase by ≥2 [13].<br>Anticholinergic load increases ≥2 [13].<br>Number of medicines contributing to each adverse drug reaction listed in the Veterans' MATES adverse event cumulative risk calculator ≥2 [28].
**Resident care record**	Record of common medicine-induced symptoms e.g., constipation, dry mouth, delirium.<br>Hospital discharge or emergency department admission in the past week.
**Activity tracker**	≥30 minutes in daily sedentary time [14, 29].
**Sleep sensor**	≥10% reduction in sleep quality; ≥1 hour change in sleep time [30, 31].
**Blood pressure**	≥10 mmHg change in systolic; ≥5 mmHg in diastolic blood pressure [32].
**Weight**	≥5% change in weight [33].
**Blood glucose level**	≥10% change.

For peer review only

Note. mmHg= millimetres of mercury.

Phase two

The pre-post feasibility study will be conducted over 12-weeks. The study will be reported according to the CONSORT checklist for pilot and feasibility trials [34]. The sleep sensor (Fibion Emfit) [19] will be installed in the rooms of all enrolled and consenting residents, and study staff will provide residents with an activity tracker (Fibion SENS) [18]. Two weeks of baseline data will be collected from these technologies prior to the start of the pharmacist service.

Pharmacist service

The on-site pharmacist will monitor the participants using the TCC-ADEPT dashboard which includes data from the digital technologies as well as daily uploaded clinical and medicine data. Where clinically significant changes are detected, the resident will be automatically flagged for the pharmacist to take appropriate action (e.g., continue monitoring, perform a review, or initiate multidisciplinary review).

Sample size

For peer review only

A sample size of 30 to 50 participants is recommended for pilot and feasibility studies [35]. The current study aims to recruit 30 participants from a 120-bed residential aged care facility.

Outcomes and analysis

Study outcomes will be measured at 4-, 8-, and 12-weeks post-baseline period. Health outcomes include number of medication adverse events and change in resident symptom score (ESAS); surrogate measure outcomes include change in physical activity and sleep; process measure outcomes include number of pharmacist recommendations provided, record of staff time spent during study implementation period, and proportion of all pharmacists' recommendations to GP's and aged care staff that are implemented.

Process evaluation to assess the feasibility of the pharmacist service

A mixed methods evaluation, applying the RE-AIM evaluation framework [36] will be performed to assess the feasibility of the pharmacist service. Descriptive statistics will be employed to report demographic characteristics and health information obtained from the Resident Care Assessment Record.

For peer review only

To determine *reach*, the number and proportion of eligible residents who were willing to participate in the study will be calculated. Information that will be collected include number of residents approached by the study staff or pharmacist and provided with the study information, number of residents who meet the inclusion criteria, number of residents who provided consent, and number of participants who completed the 12-week study.

To determine *effectiveness*, changes in resident symptom score pre- and post-digital pharmacist service, as measured using the Edmonton Symptom Assessment System (ESAS), will be assessed. Changes in medication use will be assessed, including changes to sedative and anticholinergic load pre- and post-digital pharmacist service. Changes in 24-hour movement behaviour, as measured using the activity tracker, will be assessed pre- and post-digital pharmacist service. The number of adverse events including falls (both injurious and non-injurious), fractures, delirium, faecal impaction, hospitalisation or emergency department presentation due to adverse medication events, and medication incidents will be assessed. The number of adverse events experienced by residents within the 12-weeks pre-digital pharmacist service versus number of adverse events experienced during the 12-week digital pharmacist service period will be compared. Pre-post paired t-test or Wilcoxon tests will be utilised for continuous data and McNemar's tests for categorical variables.

For peer review only

To assess service *adoption*, the number and proportion of organisation staff invited, that agreed to participate, will be calculated.

To assess *implementation*, the number and types of pharmacist recommendations will be recorded and evaluated. In addition, the proportion of recommendations made by the pharmacist will be compared with the number of recommendations accepted, to determine the acceptability of the digital pharmacist service by the GPs and aged care staff. Semi-structured interviews or focus groups will be conducted with the project pharmacist, GPs, aged care staff, residents and family members to understand their views of the digital pharmacist service, including facilitators and barriers to its delivery. An analysis will be undertaken of the costs associated with implementing the ADEPT-assisted pharmacist service. The resource use associated with the implementation of the ADEPT pharmacist service, including staff (aged care and health professionals) time, equipment, and resources will be recorded by trial staff prospectively. Changes in pharmacist time spent reviewing residents will be measured using pharmacist's record of amount of time spent reviewing medications for residents, generating recommendations and communicating with staff, GPs and residents. Resources will be valued using appropriate unit costs drawn from organisational finance data, relevant published employment awards [37], the Medicare Benefits Schedule (list of medical services that the Australian Government provides a rebate

For peer review only

to assist with costs) and the Pharmaceutical Benefits Scheme (Australian Government program that subsidises costs of prescription medications) [38, 39]. Where valuations are unable to be obtained from published data, we will value using market rates, using the average cost from three suppliers. Prices will be updated to the current year using the Consumer Price Index [40]. A budget impact analysis will be conducted, extrapolating the within-study assessment of costs to a wider population level.

To assess *maintenance*, interviews with senior management at the aged care facility will be conducted to understand intention to continue to implement the service post study funding, and whether the service aligns to the facility's mission or sustainability of business model. The capacity of the aged care organisation to implement the service (beyond the feasibility study) to identify and negate potential medicine-induced harms will be explored.

Informed consent

Prior to the conduct of any study procedures, study staff will provide residents or an authorised representative with the participant information sheet and consent form. The purpose of the study and all study procedures will be discussed with the resident or an authorised representative. Informed written consent will be obtained by the resident or

For peer review only

authorised representative. Participants will be assured that participation is completely voluntary and that they can withdraw from participation at any time.

Data management

All identifying information will be kept in secure settings in the residential aged care facility, University of South Australia (QUMPRC) or University of New South Wales, accessible only by central study staff. Where necessary, electronic tablets used for data collection (under password protection), and hard copies of questionnaires, will be stored temporarily within the residential aged care facility. The electronic tablets and forms will be stored in locked cabinets within the facility (accessible only by study staff). Information containing identifiers will be removed at the end of data collection by the project lead. The QUMPRC physical and IT environment operates to in-confidence and protected levels according to Department of Defence security standards.

Risks

The aim of the current study is to test the feasibility of using digital technologies to help pharmacists better monitor the effects of medicines that the residents are taking. There

For peer review only

are no apparent risks to participants that would be expected as a result of the pharmacist service or the unintrusive activity and sleep monitors. It is anticipated that there may be direct benefits to the participants through a reduction in adverse events.

Ethics and dissemination

Ethical approval has been obtained from the University of South Australia Human Research Ethics Committee (HREC205098). The study will be conducted in accordance with principles of the Declaration of Helsinki [41] and the National Statement of Ethical Conduct in Human Research [42]. During the study, any amendment to the study protocol or related documents will be submitted to the ethics committees for approval prior to implementation.

A Clinical Study Report in accordance with relevant ICH guidelines will be prepared, summarising all study data and analysis. Study findings may also be published or presented at scientific meetings. All publications arising from the study will comply with recognised ethical standards concerning publications and authorships. In all instances, dissemination of the study results will include de-identified or average study data and will not identify an individual participant.

For peer review only

Data availability

Individual participant data will not be made available due to data being collected from a small group of aged care residents located at one facility in South Australia, and the high risk of re-identification of individual participants.

Trial Registration number

ACTRN12623000506695

Authors' Contributions

PB, TM, DD, RM, EER and RL conceived the study and obtained study funding. MSB, PB, JS, DD, TM and RL assisted in the development of the study protocol. MSB drafted the protocol manuscript and co-ordinates the day-to-day operation of the study. PB developed the TeleClinical Care dashboard for the ADEPT study based on designs and prior development by NHL and PB. All authors critically revised and approved the final manuscript.

For peer review only

Funding statement

This project is funded by an Aged Care Research & Industry Innovation Australia (ARIIA) grant (GO00039). JS is employed by the organisation providing aged care service to the study participants. DD is supported by a Discovery Early Career Research Award (DE230101174). RL is supported by National Health and Medical Research Council Early Career Fellowship (APP1156368).

Competing interests statement

None declared.

Patient consent for publication

Not required.

For peer review only

References

1. Sluggett, J.K., et al., *Medication management policy, practice and research in Australian residential aged care: Current and future directions.* Pharmacological Research, 2017. **116**: p. 20-28.
2. Lim, R., et al., *The extent of medication-related hospital admissions in Australia: A review from 1988 to 2021.* Drug Safety, 2022. **45**(3): p. 249-257.
3. Handler, S.M., et al., *Epidemiology of medication-related adverse events in nursing homes.* The American Journal of Geriatric Pharmacotherapy, 2006. **4**(3): p. 264-272.
4. Al-Jumaili, A.A. and W.R. Doucette, *A systems approach to identify factors influencing adverse drug events in nursing homes.* Journal of the American Geriatrics Society, 2018. **66**(7): p. 1420-1427.
5. Tenhunen, M.L., B. Smithers, and B. Tucker, *Identifying medication-related adverse drug events in nursing facilities in east Texas.* The Consultant Pharmacist, 2016. **31**(8): p. 436-439.
6. Kalisch Ellett, L.M., et al., *Prevalence and Preventability of Adverse Medicine Events in a Sample of Australian Aged-Care Residents: A Secondary Analysis of Data from the ReMInDAR Trial.* Drug Safety, 2023. **46**(5): p. 493-500.
7. Sluggett, J.K., et al., *Provision of a comprehensive medicines review is associated with lower mortality risk for residents of aged care facilities: a retrospective cohort study.* Age and Ageing, 2022. **51**(7).
8. Australian Government Department of Health. *Aged care on-site pharmacist.* 2023; Available from: https://www.health.gov.au/our-work/aged-care-on-site-pharmacist.
9. Roughead, E.E., et al., *Effect of an ongoing pharmacist service to reduce medicine-induced deterioration and adverse reactions in aged-care facilities (nursing homes): a*

For peer review only

multicentre, randomised controlled trial (the ReMInDAR trial). Age and Ageing, 2022. **51**(4).

10. Lim, R., et al., *Reducing medicine-induced deterioration and adverse reactions (ReMInDAR) trial: study protocol for a randomised controlled trial in residential aged-care facilities assessing frailty as the primary outcome.* 2020. **10**(4): p. e032851.
11. Lim, R., et al., *Pharmacists as patient advocates: A series of case studies illustrating the impacts of a regular pharmacist service in residential aged care (nursing homes).* Exploratory Research in Clinical and Social Pharmacy, 2023. **10**: p. 100268.
12. Pratt, N.L., et al., *Association between use of multiple psychoactive medicines and hospitalization for falls: retrospective analysis of a large healthcare claim database.* Drug Safety, 2014. **37**(7): p. 529-35.
13. Lim, R., et al., *Using wrist-worn accelerometers to identify the impact of medicines with anticholinergic or sedative properties on sedentary time: A 12-month prospective analysis.* Maturitas, 2023. **172**: p. 9-14.
14. Theou, O., et al., *Association between sedentary time and mortality across levels of frailty.* Canadian Medical Assocition Journal, 2017. **189**(33): p. E1056-e1064.
15. Indraratna, P., et al., *Trials and tribulations: mHealth clinical trials in the COVID-19 pandemic.* Yearbook of Medical Informatics, 2021. **30**(1): p. 272-279.
16. Indraratna, P., et al., *A smartphone-based model of care to support patients with cardiac disease transitioning from hospital to the community (TeleClinical Care): Pilot randomized controlled trial.* Journal of Medical Internet Research, 2022. **10**(2): p. e32554.

For peer review only

17. Hui, D. and E. Bruera, *The Edmonton Symptom Assessment System 25 years later: Past, present, and future developments.* Journal of Pain Symptom Management, 2017. **53**(3): p. 630-643.

18. Fibion. *Fibion SENS*. 2020; Available from: https://sens.fibion.com/.

19. Fibion. *Fibion Emfit*. 2022; Available from: https://emfit.fibion.com/.

20. Roughead, E., et al., *Reducing harms from medicines in aged-care: findings from the ReMInDAR trial*. 2021, The University of South Australia.

21. Leecare, *P6Med*. 2023.

22. Medi-Map Group Pty Ltd, *Medi-Map*. 2023.

23. Nasreddine, Z.S., et al., *The Montreal Cognitive Assessment, MoCA: A brief screening tool for mild cognitive impairment.* Journal of the American Geriatrics Society, 2005. **53**(4): p. 695-699.

24. Ghaffari, A., et al., *Criterion calidity of linear accelerations measured with low-sampling-frequency accelerometers during overground walking in elderly patients with knee osteoarthritis.* Sensors, 2022. **22**(14): p. 5289.

25. Pedersen, B.S., et al., *Validation of two activity monitors in slow and fast walking hospitalized patients.* Rehabilitation Research and Practice, 2022. **2022**: p. 9230081.

26. Zink, M.D., et al., *Unobtrusive nocturnal heartbeat monitoring by a ballistocardiographic sensor in patients with sleep disordered breathing.* Scientific Reports, 2017. **7**(1): p. 13175.

27. Buimer, H.P., et al., *Sleep–wake monitoring of people with intellectual disability: Examining the agreement of EMFIT QS and actigraphy.* Journal of Applied Research in Intellectual Disabilities, 2023.

For peer review only

28. Veterans' MATES. *Cumulative risk calculator*. 2022; Available from: https://www.veteransmates.net.au/cumulative-risk-calculator.

29. Mankowski, R.T., et al., *Device-measured physical activity as a predictor of disability in mobility-limited older adults.* Journal of the American Geriatrics Society, 2017. **65**(10): p. 2251-2256.

30. Vincent, B.M., et al., *Sleeping time is associated with functional limitations in a national sample of older Americans.* Aging Clinical and Experimental Research, 2021. **33**(1): p. 175-182.

31. Goldman, S.E., et al., *Poor sleep is associated with poorer physical performance and greater functional limitations in older women.* Sleep, 2007. **30**(10): p. 1317-24.

32. Guzman, H., et al., *Clinically significant blood pressure reduction among participants of a lifestyle intervention improvement program strongly associated with reduced systemic inflammation in a high risk working population.* Circulation, 2014. **129**(suppl_1): p. AP391-AP391.

33. Williamson, D.A., G.A. Bray, and D.H. Ryan, *Is 5% weight loss a satisfactory criterion to define clinically significant weight loss?* Obesity 2015. **23**(12): p. 2319-20.

34. Eldridge, S.M., et al., *CONSORT 2010 statement: extension to randomised pilot and feasibility trials.* BMJ, 2016. **355**: p. i5239.

35. Sim, J. and M. Lewis, *The size of a pilot study for a clinical trial should be calculated in relation to considerations of precision and efficiency.* Journal of Clinical Epidemiology, 2012. **65**(3): p. 301-8.

36. Glasgow, R.E., et al., *RE-AIM planning and evaluation framework: Adapting to new science and practice with a 20-year review.* Frontiers in Public Health, 2019. **7**: p. 64.

For peer review only

37. Australian Government Fair Work Ombudsman. *Fair Work Ombudsman*. Available from: https://www.fairwork.gov.au/.

38. Australian Government Department of Health and Aged Care. *MBS Online contains the Medicare Benefits Schedule (MBS), a listing of the Medicare services subsidised by the Australian Government*. 2023; Available from: http://www.mbsonline.gov.au/internet/mbsonline/publishing.nsf/Content/Home.

39. Australian Government Department of health and Aged Care. *Pharmaceutical Benefits Scheme (PBS)*. 2023; Available from: https://www.pbs.gov.au/pbs/home.

40. Australian Bureau of Statistics. *Price indexes and inflation*. 2023.

41. World Medical Association, *World medical association declaration of Helsinki: Ethical principles for medical research involving human subjects.* JAMA, 2013. **310**(20): p. 2191-2194.

42. National Health and Medical Research Council. *National Statement of Ethical Conduct in Human Research.* 2007; Available from: https://www.nhmrc.gov.au/about-us/publications/national-statement-ethical-conduct-human-research-2007-updated-2018.

Figure 1A shows example vital signs (blood pressure, body weight, and oxygen saturation) with onset of a new medication (Alprazolam 0.25 mg tds) overlaid in the TCC-ADEPT platform. Figure 1B shows example cumulative risks and type of adverse drug reactions displayed in the TCC-ADEPT platform.

Figure 2. Proposed service flow

For peer review only

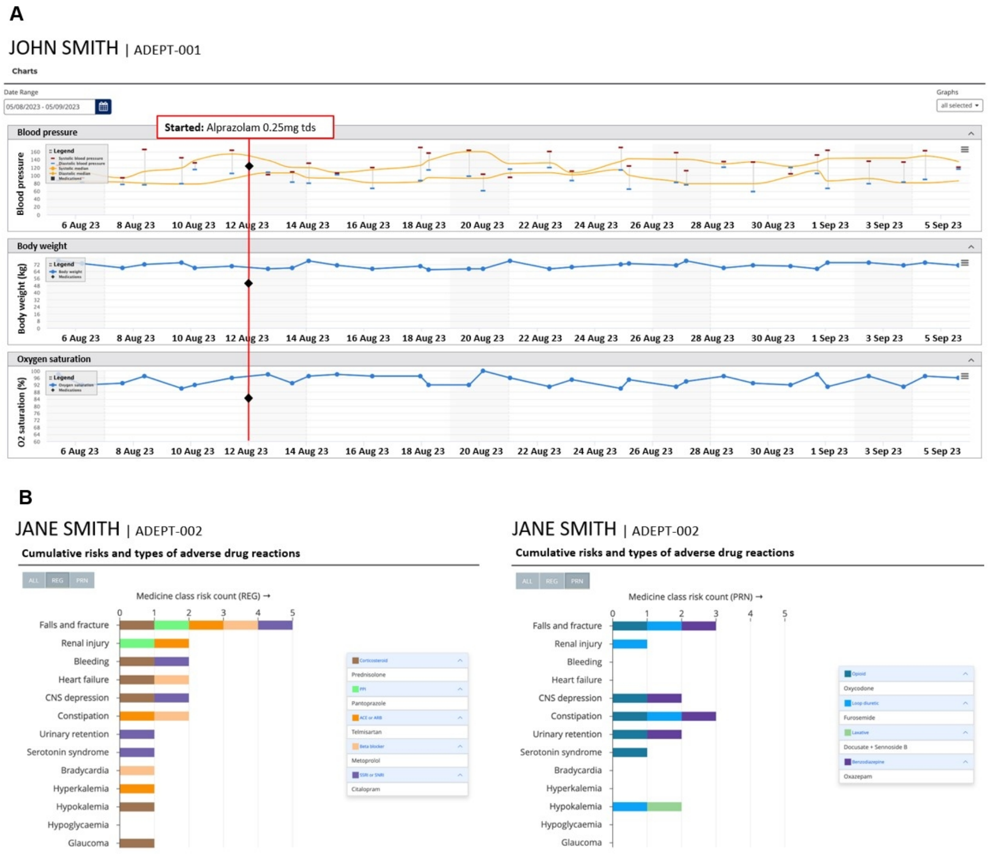

Figure 1A shows example vital signs (blood pressure, body weight, and oxygen saturation) with onset of a new medication (Alprazolam 0.25 mg tds) overlaid in the TCC-ADEPT platform. Figure 1B shows example cumulative risks and type of adverse drug reactions displayed in the TCC-ADEPT platform.

223x194mm (144 x 144 DPI)

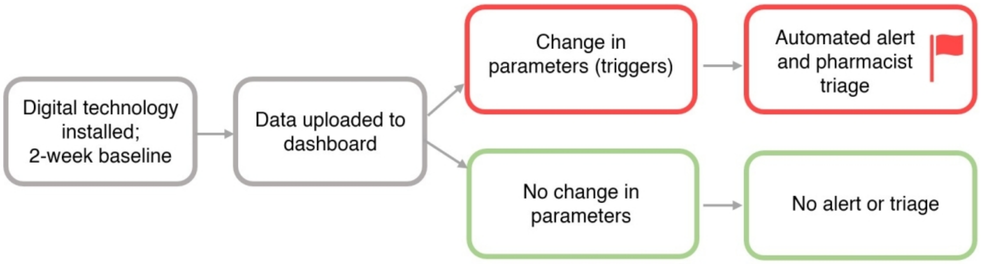

Figure 2. Proposed service flow

277x81mm (144 x 144 DPI)

University of South Australia

Consent Form

This project has been approved by the University of South Australia's Human Research Ethics Committee. If you have any ethical concerns about the project, or questions about your rights as a participant, please contact the Executive Officer of this Committee, Tel: +61 8 8302 6330; Email: humanethics@unisa.edu.au

SECTION 1: CONTACT AND PROJECT DETAILS	
Researcher's Full Name	[Chief Investigator]
Contact Details	[Phone number]
Project Number	ADEPT-2022.1.0 (Ethics ID 205098)
Project Title	A Digitally Enabled, Pharmacist service to detecT medicine harms in residential aged care: the ADEPT project

SECTION 2: PARTICIPANT CERTIFICATION

In signing this form, I confirm that:

- I have read the Participant Information Sheet, and the nature and the purpose of the research project has been explained to me. I understand and agree to take part.
- I understand the nature of my involvement in the project.
- I understand that I may not directly benefit from taking part in the project.
- I understand that I can withdraw from the project at any stage and that this will not affect my status now or in the future.
- I understand that data will be stored in the secure settings in the Quality Use of Medicines and Pharmacy Research Centre, University of South Australia. I understand that all paper and electronic records collected as part of the project will be kept for 7 years after final publication of project results.
- I understand that while information gained during the project may be published, I will not be identified and my personal results will remain confidential, unless required by law.
- I understand that data collected from this project may be used for another purpose by the researcher for which ethics approval will be sought.

Participant's Signature	*Printed Name*	*Date*
Legal guardian's Signature (if signing of behalf of participant)	*Printed Name*	*Date*

SECTION 3: RESEARCHER CERTIFICATION

I have explained the study to the participant and/or legal guardian and consider that he/she understand what is involved.

Researcher Signature	*Printed Name*	*Date*

For peer review only - http://bmjopen.bmj.com/site/about/guidelines.xhtml

SPIRIT
STANDARD PROTOCOL ITEMS: RECOMMENDATIONS FOR INTERVENTIONAL TRIALS

SPIRIT 2013 Checklist: Recommended items to address in a clinical trial protocol and related documents*

Section/item	Item No	Description	Addressed on page number
**Administrative information**			
Title	1	Descriptive title identifying the study design, population, interventions, and, if applicable, trial acronym	______1______
Trial registration	2a	Trial identifier and registry name. If not yet registered, name of intended registry	______3______
	2b	All items from the World Health Organization Trial Registration Data Set	_____N/A_____
Protocol version	3	Date and version identifier	_____N/A_____
Funding	4	Sources and types of financial, material, and other support	_____21______
Roles and responsibilities	5a	Names, affiliations, and roles of protocol contributors	____1 & 20____
	5b	Name and contact information for the trial sponsor	_____21______
	5c	Role of study sponsor and funders, if any, in study design; collection, management, analysis, and interpretation of data; writing of the report; and the decision to submit the report for publication, including whether they will have ultimate authority over any of these activities	_____N/A_____
	5d	Composition, roles, and responsibilities of the coordinating centre, steering committee, endpoint adjudication committee, data management team, and other individuals or groups overseeing the trial, if applicable (see Item 21a for data monitoring committee)	_____N/A_____

For peer review only

Introduction

Background and rationale	6a	Description of research question and justification for undertaking the trial, including summary of relevant studies (published and unpublished) examining benefits and harms for each intervention	____4 & 5_____
	6b	Explanation for choice of comparators	______19_____
Objectives	7	Specific objectives or hypotheses	____5 & 6_____
Trial design	8	Description of trial design including type of trial (eg, parallel group, crossover, factorial, single group), allocation ratio, and framework (eg, superiority, equivalence, noninferiority, exploratory)	______6______

Methods: Participants, interventions, and outcomes

Study setting	9	Description of study settings (eg, community clinic, academic hospital) and list of countries where data will be collected. Reference to where list of study sites can be obtained	______7______
Eligibility criteria	10	Inclusion and exclusion criteria for participants. If applicable, eligibility criteria for study centres and individuals who will perform the interventions (eg, surgeons, psychotherapists)	______7______
Interventions	11a	Interventions for each group with sufficient detail to allow replication, including how and when they will be administered	___11 – 14____
	11b	Criteria for discontinuing or modifying allocated interventions for a given trial participant (eg, drug dose change in response to harms, participant request, or improving/worsening disease)	_____17______
	11c	Strategies to improve adherence to intervention protocols, and any procedures for monitoring adherence (eg, drug tablet return, laboratory tests)	____18 & 19____
	11d	Relevant concomitant care and interventions that are permitted or prohibited during the trial	______7______
Outcomes	12	Primary, secondary, and other outcomes, including the specific measurement variable (eg, systolic blood pressure), analysis metric (eg, change from baseline, final value, time to event), method of aggregation (eg, median, proportion), and time point for each outcome. Explanation of the clinical relevance of chosen efficacy and harm outcomes is strongly recommended	___15 – 17____
Participant timeline	13	Time schedule of enrolment, interventions (including any run-ins and washouts), assessments, and visits for participants. A schematic diagram is highly recommended (see Figure)	____7 – 10____

For peer review only - http://bmjopen.bmj.com/site/about/guidelines.xhtml

Sample size	14	Estimated number of participants needed to achieve study objectives and how it was determined, including clinical and statistical assumptions supporting any sample size calculations	_____15______
Recruitment	15	Strategies for achieving adequate participant enrolment to reach target sample size	____7 & 15_____

Methods: Assignment of interventions (for controlled trials)

Allocation:

Sequence generation	16a	Method of generating the allocation sequence (eg, computer-generated random numbers), and list of any factors for stratification. To reduce predictability of a random sequence, details of any planned restriction (eg, blocking) should be provided in a separate document that is unavailable to those who enrol participants or assign interventions	_____N/A_____
Allocation concealment mechanism	16b	Mechanism of implementing the allocation sequence (eg, central telephone; sequentially numbered, opaque, sealed envelopes), describing any steps to conceal the sequence until interventions are assigned	_____N/A_____
Implementation	16c	Who will generate the allocation sequence, who will enrol participants, and who will assign participants to interventions	_____N/A_____
Blinding (masking)	17a	Who will be blinded after assignment to interventions (eg, trial participants, care providers, outcome assessors, data analysts), and how	_____N/A_____
	17b	If blinded, circumstances under which unblinding is permissible, and procedure for revealing a participant's allocated intervention during the trial	_____N/A_____

Methods: Data collection, management, and analysis

Data collection methods	18a	Plans for assessment and collection of outcome, baseline, and other trial data, including any related processes to promote data quality (eg, duplicate measurements, training of assessors) and a description of study instruments (eg, questionnaires, laboratory tests) along with their reliability and validity, if known. Reference to where data collection forms can be found, if not in the protocol	___7 – 11_____
	18b	Plans to promote participant retention and complete follow-up, including list of any outcome data to be collected for participants who discontinue or deviate from intervention protocols	_____N/A_____

Data management	19	Plans for data entry, coding, security, and storage, including any related processes to promote data quality (eg, double data entry; range checks for data values). Reference to where details of data management procedures can be found, if not in the protocol	___11 & 17____
Statistical methods	20a	Statistical methods for analysing primary and secondary outcomes. Reference to where other details of the statistical analysis plan can be found, if not in the protocol	___15 – 17____
	20b	Methods for any additional analyses (eg, subgroup and adjusted analyses)	_____N/A_____
	20c	Definition of analysis population relating to protocol non-adherence (eg, as randomised analysis), and any statistical methods to handle missing data (eg, multiple imputation)	_____N/A_____
**Methods: Monitoring**			
Data monitoring	21a	Composition of data monitoring committee (DMC); summary of its role and reporting structure; statement of whether it is independent from the sponsor and competing interests; and reference to where further details about its charter can be found, if not in the protocol. Alternatively, an explanation of why a DMC is not needed	_____N/A_____
	21b	Description of any interim analyses and stopping guidelines, including who will have access to these interim results and make the final decision to terminate the trial	_____N/A_____
Harms	22	Plans for collecting, assessing, reporting, and managing solicited and spontaneously reported adverse events and other unintended effects of trial interventions or trial conduct	_____19______
Auditing	23	Frequency and procedures for auditing trial conduct, if any, and whether the process will be independent from investigators and the sponsor	_____N/A_____
**Ethics and dissemination**			
Research ethics approval	24	Plans for seeking research ethics committee/institutional review board (REC/IRB) approval	_____19______
Protocol amendments	25	Plans for communicating important protocol modifications (eg, changes to eligibility criteria, outcomes, analyses) to relevant parties (eg, investigators, REC/IRBs, trial participants, trial registries, journals, regulators)	___19 – 20____

Consent or assent	26a	Who will obtain informed consent or assent from potential trial participants or authorised surrogates, and how (see Item 32)	______17_______
	26b	Additional consent provisions for collection and use of participant data and biological specimens in ancillary studies, if applicable	______N/A______
Confidentiality	27	How personal information about potential and enrolled participants will be collected, shared, and maintained in order to protect confidentiality before, during, and after the trial	_____17 & 20_____
Declaration of interests	28	Financial and other competing interests for principal investigators for the overall trial and each study site	______21_______
Access to data	29	Statement of who will have access to the final trial dataset, and disclosure of contractual agreements that limit such access for investigators	______17_______
Ancillary and post-trial care	30	Provisions, if any, for ancillary and post-trial care, and for compensation to those who suffer harm from trial participation	______N/A______
Dissemination policy	31a	Plans for investigators and sponsor to communicate trial results to participants, healthcare professionals, the public, and other relevant groups (eg, via publication, reporting in results databases, or other data sharing arrangements), including any publication restrictions	______20_______
	31b	Authorship eligibility guidelines and any intended use of professional writers	______20_______
	31c	Plans, if any, for granting public access to the full protocol, participant-level dataset, and statistical code	______20_______
**Appendices**			
Informed consent materials	32	Model consent form and other related documentation given to participants and authorised surrogates	supplemental material
Biological specimens	33	Plans for collection, laboratory evaluation, and storage of biological specimens for genetic or molecular analysis in the current trial and for future use in ancillary studies, if applicable	______N/A______

*It is strongly recommended that this checklist be read in conjunction with the SPIRIT 2013 Explanation & Elaboration for important clarification on the items. Amendments to the protocol should be tracked and dated. The SPIRIT checklist is copyrighted by the SPIRIT Group under the Creative Commons "Attribution-NonCommercial-NoDerivs 3.0 Unported" license.

For peer review only - http://bmjopen.bmj.com/site/about/guidelines.xhtml
